# Pneumococcal Serotype Evolution and Burden in European Adults in the Last Decade: A Systematic Review

**DOI:** 10.3390/microorganisms11061376

**Published:** 2023-05-24

**Authors:** Rita Teixeira, Vasiliki Kossyvaki, Paulina Galvez, Cristina Méndez

**Affiliations:** 1 Vaccines and Antivirals Department, Pfizer Portugal, 1300-477 Lisbon, Portugal; 2 Vaccines Department, Pfizer Greece, 10431 Athens, Greece; 3 Vaccines and Antivirals Department, Pfizer Spain, 28108 Madrid, Spain

**Keywords:** pneumococcal disease, PCV13, PCV15, PCV20, pneumococcal vaccination, public health, systematic review, epidemiology, adults, Europe

## Abstract

Pneumococcal disease is a major cause of morbidity/mortality worldwide, and vaccination is an important measure in its prevention. Despite European children being vaccinated with pneumococcal conjugate vaccines (PCVs), pneumococcal infections are still a major cause of morbidity/mortality in adults with risk conditions and their vaccination might be an important prevention strategy. New PCVs have been approved, but information is lacking on their potential impact in European adults. In our review, we searched PubMed, MEDLINE, and Embase for studies on the additional PCV20 serotypes (concerning incidence, prevalence, disease severity, lethality, and antimicrobial resistance) in European adults, between January 2010 and April 2022, having included 118 articles and data from 33 countries. We found that these serotypes have become more prevalent in both invasive and non-invasive pneumococcal disease (IPD and NIPD), representing a significant proportion of cases (serotypes 8, 12F, 22F) and more serious disease and/or lethality (10A, 11A, 15B, 22F), showing antimicrobial resistance (11A, 15B, 33F), and/or affecting more vulnerable individuals such as the elderly, immunocompromised patients, and those with comorbidities (8, 10A, 11A, 15B, 22F). The relevance of pneumococcal adult carriers (11A, 15B, 22F, and 8) was also identified. Altogether, our data showed an increase in the additional PCV20 serotypes’ prevalence, accounting for a proportion of approximately 60% of all pneumococcal isolates in IPD in European adults since 2018/2019. Data suggest that adults, as older and/or more vulnerable patients, would benefit from vaccination with higher-coverage PCVs, and that PCV20 may address an unmet medical need.

## 1. Introduction

*Streptococcus pneumoniae* (pneumococcus) causes severe bacterial infections in humans, manifesting either as invasive pneumococcal disease (IPD, such as bacteremic pneumonia, bacteremia, and meningitis) or non-invasive pneumococcal disease (NIPD, such as acute otitis media, sinusitis, and non-bacteremic pneumonia) [1]. Pneumococcal disease is a major cause of morbidity and mortality worldwide, having caused 197 million episodes of pneumococcal lower respiratory tract infection and 1.2 million deaths in 2016 [2]. Disease burden (including higher incidence and mortality rates [2]) is greatest among children under 5 years and adults over 65 years old, as well as in individuals with comorbidities, such as immunosuppression or chronic medical conditions [3,4,5].

The capsular polysaccharide of the pneumococcus is its main virulence factor, and more than 90 different capsular serotypes exist. These serotypes differ not only in their structure but also in the disease severity and case fatality they are associated with, as well as their invasiveness, antimicrobial susceptibility, and geographical areas and age groups they affect [6,7,8,9]. These serotypes are also the basis of the current pneumococcal vaccines. Vaccination is the most effective public health measure in the prevention of pneumococcal disease, being part of many European national immunization programs (NIPs) for children [6]. Pediatric vaccination reduces nasopharyngeal carriage of the vaccinal serotypes, preventing transmission to under- or unimmunized children and adults, resulting in herd protection and overall disease reduction in all ages [9,10,11]. However, pneumococcal infections are still a major cause of morbidity and mortality in adults over 65 years old and/or with risk conditions, with a significant proportion of infections being caused by vaccinal serotypes [9], suggesting that pediatric immunization alone results in indirect protection, important, albeit insufficient to protect this age group. Moreover, some of the most prevalent serotypes (both vaccinal and non-vaccinal) often differ between children and adult populations [12,13]. Thus, adult pneumococcal vaccination might be an important prevention strategy against pneumococcal disease. Pneumococcal vaccination is currently recommended in some countries for certain groups of adults at increased risk of pneumococcal disease, including those over 65 years and immunocompromised individuals [4,5,6,8,14]. Nonetheless, vaccination in this age group remains suboptimal [15].

Currently, two types of vaccines have been approved for the prevention of pneumococcal disease: a 23-valent pneumococcal polysaccharide vaccine (PPSV23) and pneumococcal conjugate vaccines (PCVs). The PPSV23 includes 23 purified capsular polysaccharide antigens (serotypes: 1, 2, 3, 4, 5, 6B, 7F, 8, 9N, 9V, 10A, 11A, 12F, 14, 15B, 17F, 18C, 19A, 19F, 20, 22F, 23F, and 33F) and was licensed in the United States of America (US) in 1983 [16]. However, polysaccharide vaccines primarily induce B-cell-dependent immune responses, limited in being long-lasting or anamnestic upon subsequent challenge with native polysaccharides [17]. In order to stimulate a substantial immune response, vaccines conjugating the pneumococcal capsular polysaccharides from frequent serotypes with a highly immunogenic protein, such as a non-toxic diphtheria toxoid (CRM197), were generated. These vaccines (PCVs—pneumococcal conjugate vaccines) induce a B- and T-cell response, resulting in mucosal immunity and effective protection against vaccine serotypes, thereby reducing carrier rates and pneumococcal disease [16,17].

The first PCV, PCV7 (covering serotypes: 4, 6B, 9V, 14, 18C, 19F, and 23F), was licensed in 2000–2001 and was integrated into the NIPs of many European countries between 2006 and 2008. Pediatric immunization with PCV7 prevented pneumococcal disease caused by the vaccinal serotypes as well as colonization and transmission to adults, leading to an overall reduction in disease burden in all ages [18]. IPD incidence due to PCV7 serotypes became very low in all age groups and almost absent in children, particularly in countries with high vaccination rates [12]. However, a few years after PCV7, the epidemiology of pneumococcal disease changed and non-vaccinal serotypes emerged as the most prevalent ones, in a phenomenon designated ‘serotype replacement’ [19]. Two higher-valency PCVs, PCV10 (covering PCV7 serotypes plus serotypes: 1, 5, and 7F) and PCV13 (covering PCV7 serotypes plus: 1, 3, 5, 6A, 7F, and 19A), replaced PCV7 in pediatric vaccination in Europe between 2009 and 2011 [20]. Data collected over the past decade suggest that serotype replacement has reoccurred, and that new vaccines will be necessary to prevent pneumococcal disease caused by emergent serotypes, such as 8, 12F, and 22F [13,21]. In light of this increase in the prevalence of non-vaccinal serotypes, the new PCV15 (covering the additional serotypes: 22F, and 33F) and PCV20 (covering the additional serotypes: 8, 10A, 11A, 12F, 15B, 22F, and 33F) have recently been approved for prevention of pneumococcal disease in individuals aged 18 years old and above in Europe and the US [22,23,24], being likely to replace PCV10 and PCV13 in NIPs over time, if also approved for use in children [6].

The currently available PCV of highest valency, PCV20 (comprising all serotypes included in recent PCVs), has been found to be safe and well tolerated, eliciting robust immunologic responses (including OPA—opsonophagocytic antibody responses) for its 20 serotypes one month after vaccination, in adults aged 18–49, 50–59, 60 years and above [25], and over 65 years previously vaccinated with other pneumococcal vaccine regimens [26].The 7 additional serotypes of PCV20 were selected based on their high prevalence as a cause of disease and pneumonia, their generalized geographical distribution, and/or other factors, such as association with antibiotic non-susceptibility (serotypes: 11A, 15B), outbreaks (serotypes: 8, 12F), and more severe disease or increased mortality rate (serotypes: 10A, 11A, 22F) [27].

In this review, we have focused on the evolution of the prevalence and impact (incidence, burden, lethality, and antibiotic resistance) of each additional PCV20 serotype (which also include the additional PCV15 serotypes) in European adults. This review has focused on PCV20′s added serotypes’ clinical and epidemiological value to help support future decision making on adult pneumococcal vaccination policies.

## 2. Methodology

This systematic review was conducted in accordance with the Preferred Reporting Items for Systematic Reviews and Meta-Analyses (PRISMA) reporting guidelines [28].

### 2.1. Search Strategy

The PubMed and MEDLINE databases were searched for relevant studies published between January 2010 and April 2022. Only articles written in English and peer-reviewed studies from indexed journals were considered. Several searches were conducted in order to determine the best search strategy (Table S1). The ultimate searches included a combination of MeSH and free search terms and were as follows: “Pneumococcal Infections [MeSH Major Topic] AND Europe [MeSH Terms] AND (adult) AND (serotype)”, “Europe [MeSH Terms] AND (adult) AND (pneumococcal serotype) NOT Pneumococcal Infections [MeSH Major Topic]”, “Europe [MeSH Terms] AND (adult) AND (pneumococcal serotype 8) NOT Pneumococcal Infections [MeSH Major Topic]”, and “Europe [MeSH Terms] AND (adult) AND (pneumococcal serotype 11A) NOT Pneumococcal Infections [MeSH Major Topic]”. Corresponding Emtree terms were used in Embase searches. Searches were designed to maximize the information retrieved while also avoiding its duplication/overlap (see Table S1).

### 2.2. Inclusion Criteria and Study Selection

This systematic review investigated the evolution of the prevalence and impact of serotypes 8, 10A, 11A, 12F, 15B, 22F, and 33F on pneumococcal disease, both invasive and non-invasive, in European adult populations (aged 18 to 64 years and aged 65 years and over). This review included studies published in the past decade (January 2010 to April 2022), including (but not limited to) observational studies, such as cross-sectional studies, prospective and retrospective comparative cohort studies, time-series studies, case–control studies, and randomized controlled trials. Reviews and systematic reviews that contained data from European national registries on pneumococcal disease were also included, provided their data did not overlap with other studies already included.

Studies were excluded if at least one of the following was applicable: having no mention of at least one of the serotypes of interest (8, 10A, 11A, 12F, 15B, 22F, 33F); not addressing epidemiological data on these serotypes (such as incidence, prevalence, burden, lethality, antibiotic resistance); being performed in non-European populations; focusing exclusively on children; or being published before 2010. Studies on mixed populations (e.g., all age groups—5–64 years, etc.) were included, being signalized as such in the results.

Study selection was performed by two different reviewers who screened the titles and abstracts from the retrieved articles. If an abstract or title did not contain sufficient information to determine eligibility, the full text was reviewed. The reviewers were not blinded to each other’s decisions. Disagreements were resolved by discussion or with the assistance of a third reviewer.

### 2.3. Outcome Measures

The outcomes of this review included pneumococcal disease incidence, burden, and mortality associated with the additional PCV20 serotypes, as well as serotype prevalence, lethality, carriage and resistance to antimicrobials. The outcomes also corresponded to a certain time frame, which altogether was used to study the evolution of the additional PCV20 serotypes over time.

### 2.4. Data Extraction and Synthesis

**Figure 1:**
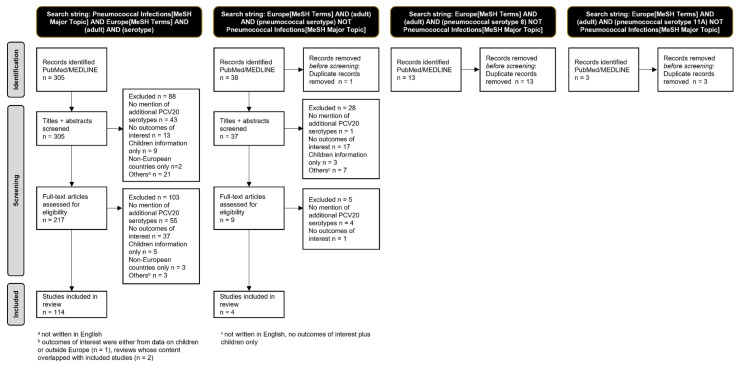
PRISMA flow diagram for literature review process and selected studies.

The number of studies in each step of the process of study selection was represented in a PRISMA flow diagram (Figure 1). Full-text articles that remained eligible for inclusion were read by the investigators. Key study information such as the reference, country, timeframe, population(s), pneumococcal disease studied (i.e., IPD, CAP—community-acquired pneumonia), additional PCV20 serotypes reported, and outcomes of interest were inserted into supplementary tables (Supplementary Tables S2–S5).

These studies were also divided by timeframe according to the PCVs licensure dates and the PCV in use, in order to stratify collected data: pre-PCV7 (before 2001, Supplementary Table S2), PCV7 (2001–2009, Supplementary Table S3), PCV10/13 (2010–2015, Supplementary Table S4), and post-PCV10/13 (2016–2022, Supplementary Table S5). If data corresponded to more than one timeframe, the outcomes were included in both tables. In some cases, the PCV era was adjusted if the individual study defined one of these periods with different dates.

Each study’s internal validity was assessed independently by two reviewers. Disagreements were resolved by discussion or with the assistance of a third reviewer.

## 3. Results

**Figure 2:**
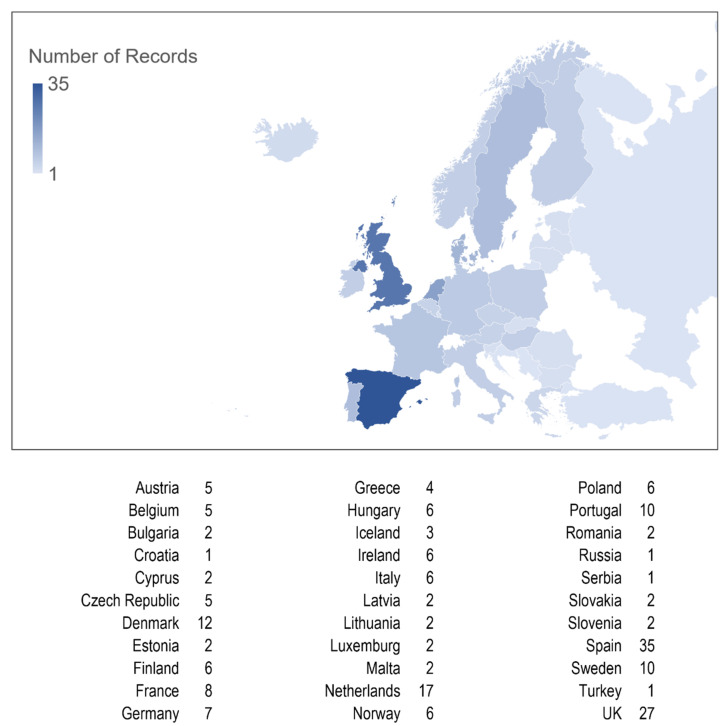
Number of records included in the study per country.

A total of 359 papers were identified and screened, and 118, containing data from 33 different European countries, were included in this systematic review (Figure 2, Figure S1).

From the included papers, 28 represented data from the pre-PCV7 era, 85 from the PCV7 era, 72 from the PCV10/13 era, and 33 from the post-PCV10/13 era (with several papers spanning two or more PCV eras). Most of the data described the incidence and/or prevalence and/or proportion of the different serotypes in IPD in different countries or regions, followed by information of the severity and lethality of each serotype and, to a lesser extent, data on the incidence and/or prevalence and/or proportion of each serotype in NIPD and antimicrobial resistance. Data from studies included in this systematic review are detailed in Supplementary Tables S2–S5, corresponding to each of the different PCV eras, and the most relevant findings are summarized in the sections below.

To enhance data visualization, we plotted the number of records in which each of the serotypes was found to be important (either due to high incidence/prevalence, representing more severe disease, or showing antimicrobial resistance) over the different PCV eras (Figure S1). Since the eras showed an uneven number of records, we divided the number of records of each serotype by the total number of records of each era and plotted it as a proportion (e.g., serotype 8 was only found to be important in 29% of the pre-PCV era studies, whereas it was important in 75% of the records from the post-PCV10/13 era).

### 3.1. Serotype Incidence and Prevalence (IPD)

IPD incidence and prevalence for each serotype varied greatly across Europe and, at times, even regions within the same country, over time. Due to the extensive data set on IPD, only the overall European proportion of each serotype over time is stated below. Detailed information on each country and individual studies are presented in Supplementary Tables S2–S5.

Serotype 8 displayed a 3–7% prevalence in IPD across Europe in the pre-PCV7 era (Supplementary Table S2). By the PCV7 era, it corresponded to 3–10% (Supplementary Table S3), with studies from some countries (such as Spain and England) showing 13.5% and 18.9% prevalence, albeit for the whole population [29,30]. In the PCV10/13 period, most countries displayed a prevalence of serotype 8 between 4 and 8% (Supplementary Table S4): data from 2014–2015 being closer to 8–10% [31,32]; its proportion in IPD being generally higher in younger versus older adults [31,33,34,35]; and being more prevalent in IPD than NIPD [36]. By the post-PCV10/13 era, serotype 8 emerged across Europe as the most prevalent serotype in IPD in adults in most countries, identified in 15–30% of the IPD isolates (Supplementary Table S5). The rapid emergence of serotype 8 among European adults (best exemplified by Spanish data where its prevalence grew from 14% to 30% in adults aged 18–64 and 8.9% to 19% in adults ≥65 years [37]) is worth concern.

Serotype 10A was identified in approximately 1–2% of the IPD isolates in the pre-PCV7 era (Supplementary Table S2), varying between 1 and 4% in the following (PCV7 and PCV10/13) eras (Supplementary Tables S3 and S4). By the post-PCV10/13 era, the prevalence for this serotype was between 2 and 5% in Europe (Supplementary Table S5). As for serotype 11A, it went from 1–2% of all IPD isolates in the pre-PCV7 era (Supplementary Table S2), to 1–4% in the PCV7 and PCV10/13 eras (Supplementary Tables S3 and S4), and 2–7% in the post-PCV10/13 era, albeit very variable between countries (Supplementary Table S5).

Serotype 12F was infrequently isolated in the pre-PCV7 era (2–3% prevalence, Supplementary Table S2), reaching 2–5% in the PCV7 era (Supplementary Table S3), and representing 3–8% of all IPD isolates in the PCV10/13 era (Supplementary Table S4)—some studies finding it more prevalent in younger than older adults [34,38,39]. By the post-PCV10/13 era, the prevalence of serotype 12F came close to 10% in several European countries (Supplementary Table S5).

Serotype 15B was identified in 1% of all IPD isolates in the pre-PCV7 era (Supplementary Table S2). In subsequent eras, it remained stable at approximately 1–3% (Supplementary Tables S3–S5).

Serotype 22F displayed a prevalence of 1–4% in the pre-PCV7 era (Supplementary Table S2), increasing to 2–6% in the PCV7 era (Supplementary Table S3). Some studies reported a significant increase in the incidence of serotype 22F between these eras [40,41,42,43,44], emerging among the most prevalent serotypes in several countries [45,46]. By the PCV10/13 era, serotype 22F was indeed identified in approximately 4–7% of all IPD isolates, although variable between countries, remaining stable into the post-PCV10/13 era (Supplementary Tables S4 and S5). Additionally, and contrary to serotypes 8 and 12F, the incidence of serotype 22F was higher in older versus younger adults, something first reported in the PCV7 era [47,48], but also in later PCV eras [31,34,35,39,48].

Lastly, serotype 33F was identified in 1–3% of IPD isolates in the pre-PCV7 and PCV7 eras (Supplementary Tables S2 and S3), 1–4% in the PCV10/13 era (Supplementary Table S4), and 2–4% in the post-PCV10/13 era (Supplementary Table S5).

### 3.2. Serotype Incidence and Prevalence (NIPD and Carriage)

Although our search included all instances of pneumococcal disease, both IPD and NIPD (and carriage studies in adults), much fewer records on NIPD (and carriage) were available in the literature. These were also more frequent in the late PCV eras (Supplementary Tables S4 and S5) and for specific serotypes, such as 11A.

Serotype 11A was found frequently in carriage, identified in 6.4% of carriers in an European study with individuals of all ages (reaching approximately 11% in France and Sweden) [49], in 8.3% of older adults that were carriers in Italy [50], in 7.7% of the carriers among young adults in the UK [51], and in 6.4% of carriers over 60 years in Portugal in the PCV10/13 era [52]. In addition to being one of the most frequent in carriage, serotype 11A was found to be significantly associated with NIPD versus IPD in Portugal (in the PCV7 and PCV10/13 eras) [53], showing 6.7% prevalence in NIPD in Portugal (adult population) [53], 5.3% in Spain (whole population) [54] and 3.9% in Greece (adult population) [55] in the PCV7 era. By the PCV10/13 era, this serotype represented 9.2% of NIPD in Portuguese adults [56] and 9.3% in Spanish adults [57]. Furthermore, a study from Mallorca (Spain) found serotype 11A in 14.3% of the isolates responsible for pneumococcal disease, but only in 3.1% of the isolates causing invasive disease, suggesting again that this serotype associates with NIPD [58]. Similar findings were published in a Danish study, in which serotype 11A had a prevalence of 7.7% in non-bacteremic pneumonia versus 1.1% in bacteremic pneumonia [36].

As for the other additional PCV20 serotypes, in the PCV10/13 and post-PCV10/13 eras, serotype 8 was sometimes found as one of the most prevalent serotypes in adults hospitalized with CAP, with prevalence rates approximately 7–8% [59,60,61], although it varied in different countries, from 1.6% in Germany [62] to 18.3% in the UK [63]. Between the PCV7 and post-PCV10/13 eras, serotype 12F was found to be an important contributor to adult CAP in some countries, such as the Netherlands, Spain, and the UK (4–6% prevalence rates) [59,63,64]. Similar data were found for serotype 22F, which showed a 3–8% prevalence rate in CAP in Spain, Belgium, the UK, Portugal, and Denmark (albeit these data refer to all age groups) [64,65]. This serotype was also among the most prevalent ones in NIPD in Portugal (4.8%) [56].

Regarding carriage, additional PCV20 serotypes were identified in a study involving several European countries with individuals of all ages [49]. Serotype 10A was found in carriage in approximately 3% (being lower in Austria, Croatia and Sweden and higher in the UK at 13.9%), serotype 15B corresponded to 3.7%, and serotype 22F comprised 3.4% [49]. More importantly, some of these serotypes were also found in carriage in adults. Serotype 15B represented 8.3% of the older adults that were carriers in Italy [50]. Serotype 22F was among the most prevalent serotypes in carriage (9%) in individuals over 60 years in Portugal in the PCV10/13 era [52]. As for serotype 8, although in England it was found to have a high case to carrier ratio (i.e., being very prevalent in IPD but not in carriage) [66], studies in young adults in the UK [51] and older adults in Italy [50] found that this serotype accounted for 7.7% and 16.7% of the carriers, respectively.

### 3.3. Serotype Associated Disease Severity and Lethality

Data on serotype associated disease severity and lethality were sometimes contradictory in the studies included in our review. When this was the case, the results we summarize aim at representing both perspectives, or, when several studies made the same claim, these results were considered instead. Detailed information on individual studies is presented in Supplementary Tables S2–S5.

Serotypes 8 and 12F were less common in a high-risk group of patients [67], and more prevalent in individuals with no comorbidities comparing to the other serotypes [38]. However, Spanish studies found serotype 8 to be more frequent among HIV-infected patients with IPD [29,31], and to be the most prevalent serotype in CAP in patients with chronic renal failure, patients with asthma, tobacco cigarette smokers, and patients with diabetes mellitus (33.3%, 14.6%, 14.2%, 8.7%) [61]. Regarding serotype 12F, it was often associated with septicemia [68] and septic arthritis [69], as well as being among the serotypes most associated with risk of ICU admission, both at admission and during hospitalization, due to CAP [61].

As previously highlighted, serotype 8 was found to have enhanced propensity to cause invasive disease—OR 46.2 [70]. On the other hand, serotypes 11A and 15B were associated with carriage, having a lower invasive disease potential [70]. Serotype 8 also showed higher incidence in younger adults versus older (15–64 vs. ≥65 years old), as did serotype 12F [38]. Concomitantly, serotypes 11A and 22F were associated with significantly older IPD patients [44,57,71].

Serotype 10A was found by multiple studies to be associated with a higher propensity for meningitis [47,72,73] and septicemia [73] but not pneumonia [34]. Serotype 15B was also more associated with meningitis and septicemia than with pneumonia [34,73], and the same was found for serotype 22F, which showed significant lower OR for pneumonia but significantly higher OR for septicemia [68,69,73,74] and meningitis [47,72]. It was also among the serotypes most associated with risk of sepsis/septic shock on admission or during hospitalization due to CAP [61].

Serotype 22F was also frequently associated with comorbidity prevalence (68.7%) as was serotype 33F (67.2%), both significantly higher than the other serotypes [71]. Serotypes 10A, 15B and 33F were also significantly more common in high-risk group patients (39%, 52% and 30% vs. 16%, respectively), associating significantly with IPD in this population [67].

Regarding the lethality associated with each serotype, serotype 11A was the one most found to associate with higher risk of mortality and/or case fatality ratio (CFR) in IPD [34,68,75,76]. Serotypes 15B and 22F were also found to be associated with higher CFR in IPD [34,75], albeit to a lesser extent. Serotype 8 associated with lower OR for death [47,72] and/or lower CFR [38,75], and so did serotype 12F [38,75]. Serotypes 10A and 22F showed a high case fatality proportion in meningitis [77].

### 3.4. Serotype Associated Antimicrobial Resistance

Serotype 11A was the most associated with antimicrobial resistance. In the PCV7 era, it represented 14.3% of the IPD isolates resistant to erythromycin in Spain [78], while representing 5.3% of the IPD isolates in Poland showing resistance to penicillin and cefotaxime [79]. Concomitantly, serotype 11A isolates from NIPD in Spain showed non-susceptibility rates of approximately 35% for several antimicrobials such as penicillin, amoxicillin, erythromycin and cefuroxime [54]. In the PCV10/13 era, serotype 11A continued to emerge as associated with antimicrobial resistance in IPD isolates, being among the most commonly showing resistance to erythromycin (in Poland [34] and Spain [31,80]) but also penicillin and cefotaxime (in Spain [31,80]). Serotype 11A was the only one in reports of antimicrobial resistance in the post-PCV10/13 era, particularly in Spain, where the proportion of penicillin- and/or penicillin and amoxicillin-resistant serotypes has been increasing [57,81].

Serotypes 15B and 33F were also often associated with antimicrobial resistance. Approximately 20% of the serotype 15B IPD isolates in Poland were found to show resistance to penicillin in the PCV7 era [79]. In the PCV10/13 era, 25% of the serotype 15B isolates in IPD in Spain were found to show erythromycin non-susceptibility [31], a proportion which was approximately 20% in Poland [34]. Serotype 33F displayed macrolide-resistant isolates in Spain in the PCV7 era [82], and was among the most frequent serotypes showing erythromycin resistance in this country both in the PCV7 and the PCV10/13 eras (12.9% and 11.6% of the isolates, respectively) [80]. This serotype also emerged in Norway as frequently displaying resistance to several antimicrobials in IPD (erythromycin, trimethoprim/sulfamethoxazole, and clindamycin), both in the PCV7 and the PCV10/13 eras [83].

Records of antimicrobial resistance in isolates from serotypes 8, 10A and 12F across Europe were uncommon throughout the past decade. Some studies in the PCV7 era found serotype 8 IPD isolates to show resistance to erythromycin [80], tetracycline, norfloxacin and erythromycin [29], and norfloxacin and levofloxacin [84], but only in Spain. In the PCV10/13 era, another Spanish study reported that 19.3% of the serotype 8 IPD isolates showed non-susceptibility to levofloxacin and 22.9% to erythromycin [31]. Only one study (also in Spain, in the PCV10/13 era) reported that 31.8% of the serotype 10A IPD isolates showed erythromycin non-susceptibility [31]. Similarly, another Spanish study (in the PCV7 era) found that 7.1% of the serotype 12F IPD isolates were resistant to penicillin and 7.1% to erythromycin [78]. No records of antimicrobial resistance were found for serotype 22F.

Detailed information on antimicrobial resistance is presented in Supplementary Tables S3–S5 (no records having been found for the pre-PCV7 era).

## 4. Discussion

Although pneumococcal disease burden in adults (particularly older adults) remains rather high, with pneumococcus leading to approximately 30% of adults’ hospitalizations due to CAP in Europe and 8–12% in the US [63], vaccination in this age group remains suboptimal [15], and some debate subsists regarding optimal vaccination policies. Moreover, the epidemiology of pneumococcal disease in Europe differs from that of the US, with CAP caused by pneumococcus representing approximately 26% in Europe versus 11% in North America [8]. In this systematic review, we aimed to assess the evolution in prevalence and the impact of the additional PCV20 serotypes over the last decade in European adult and elderly populations, in order to support efficient decision making and inform future vaccination policies.

### 4.1. Findings of This Study—The Additional PCV20 Serotypes in European Adults in the Past Decade

In this work, we found evidence showing a great increase in the prevalence of serotypes 8, 12F and 22F in IPD over the past decade in European adults. In the most recent epidemiological eras, serotype 8 showed an incidence of 15–30%, whereas serotypes 12F and 22F accounted for 7–10% each. These serotypes were also important contributors to the burden of CAP in several European countries [59,60,61,63,64,65,75].

Serotypes 8 and 12F were more prevalent in younger versus older adults [31,33,34,35,38,39], whereas serotype 22F showed a higher prevalence in the elderly population [31,34,35,39,47,48]. Additionally, serotypes 8 and 12F appeared to be less present in patients with comorbidities and associate with lower CFR [38,47,67,72,75], although some studies, particularly on immunocompromised patients and CAP, challenged this notion [29,31,61]. On the other hand, serotype 22F was more often found in patients with comorbidities [71] and has been found to be associated with septicemia [61,68,69,73,74], meningitis [47,72], and death [34,75].

Although some serotypes, particularly 8, show high propensity for causing invasive disease [70], others, such as 11A (and to a lesser extent 15B), were significantly associated with carriage and non-invasive pneumococcal disease [53,70]. Serotype 22F was found in both IPD and NIPD (and carriage). We also found evidence that serotype 11A is associated with older patients [44,57,71], antibiotic non-susceptibility [31,34,54,57,78,79,80,81], and increased risk of death [34,44,68,75,76]. Since this is a prevalent serotype (2–7% in IPD and 9–10% in NIPD, in recent years), these data are worrisome.

Despite showing lower IPD incidence rates, serotypes 10A [47,72,73] and 15B were associated with higher risk of meningitis and septicemia, but lower risk of developing pneumonia [34,73], and serotypes 15B and 33F were also found to display antimicrobial resistance [31,34,80,82,85]. Parallelly, serotype 15B was associated with higher CFR in IPD [34,75] and serotype 33F was more often found in patients with comorbidities [71]. Serotypes 10A, 15B and 33F also associated with IPD in high-risk patients [67].

Altogether, over the past decade, the additional PCV20 serotypes have shown an increase in prevalence, being associated with more serious disease, lethality, antimicrobial resistance, and/or more vulnerable individuals (elderly, immunocompromised patients, patients with comorbidities) in European adults. The emergence of PCV20 serotypes over the last decade is displayed in Figure S1, where we plotted the number of records in which each of the serotypes was found to be important (either due to high incidence/prevalence, representing more severe disease, or showing antimicrobial resistance) over the different PCV eras. It is shown in Figure S1 that the published records for PCV20 additional serotypes, particularly serotypes 8, 12F, 22F and 11A (and to a lesser extent 33F, 15B, and 10A), have increased since the pre-PCV7 and PCV7 eras, suggesting the emergence of these serotypes as important contributors to pneumococcal disease in the modern PCV eras.

### 4.2. The New Paradigm in Carrier Status: Pneumococcal Carriage in Adults

In our review, we found several studies showing carriage of the additional PCV20 serotypes in adults, namely 11A, 15B, 22F, and 8 [50,51,52], despite the latter having been reported to be low in carriage, particularly in children [66]. The nasopharynx is stated as being the main reservoir of pneumococcus, particularly in children under 5 years of age, which are considered the main dissemination vector of this pathogen in the community [3,86]—*Streptococcus pneumoniae* having indeed been detected in 60–90% of children, depending on geographic location [87,88]. Nonetheless, recent studies revealed that approximately 10% of adults aged 60–65 years are also pneumococcal carriers, and this proportion might be even higher in populations with risk factors (e.g., residing in a nursing home) [86,89], namely since data have pointed to a risk of pneumococcal colonization (at least once during a period of one year) of 58% in healthy adults [90]. An important proportion of adult carriers is likely to affect the overall reservoir of circulating pneumococcus at community levels, impacting the magnitude of herd protection provided by pediatric vaccination. Moreover, it can account for some of the serotype differences observed between populations of different ages, for example serotypes 3, 8 and 19A, which are more prevalent in adults than children, partly explaining the persistent circulation of serotypes 3 and 19A despite high pediatric vaccinal coverage [12,13,86]. The carrier status new paradigm supports pneumococcal vaccination in older adults, potentially reducing adult pneumococcus carriage and protecting certain groups of adults at increased risk of pneumococcal disease.

### 4.3. Adult Vaccination and the Additional Benefit of PCV20

Altogether, our data showed an increase in the additional PCV20 serotypes’ prevalence in European adults in the past decade. In terms of vaccinal coverage, a European study on adults found that in 2018, the serotypes included in PCV15 represented one-third of the IPD cases, whereas serotypes included in PCV20 represented two-thirds of the IPD cases [21]. This is in line with data from Spanish adults, where in 2015–2016, the proportion of PCV20 additional serotypes causing IPD was 63.3% [91], remaining at 62% for adults ≥65 years in 2019 [37], and with a German study in which the coverage of PCV20 against pneumococcal CAP in adults was 63.2% (versus 31.6% for PCV13 and 36.8% for PCV15) [62]. Although less data were available for NIPD, a study from Spain on CAP in adults found that in 2017–2018, CAP due to *Streptococcus pneumoniae* was caused by 34.9% of the PCV13 serotypes, 36.1% of the PCV15 serotypes and 79.5% of the PCV20 serotypes in adults aged 18–64 years old, and 40.2%, 46.4% and 66% for those aged 65 years and up, respectively [61].

The US Advisory Committee on Immunization Practices (ACIP) has, since October 2021, recommended the use of either PCV20 alone or PCV15 in series with PPSV23 for all adults aged ≥65 years, and for adults aged 19–64 years with certain underlying medical conditions or other risk factors who have not previously received a PCV or whose previous vaccination history is unknown [22]. Although both immunization strategies were found to be immunogenic, safe, and cost-effective [22], a previous study in adults over 65 years old in the US, taking into account the pneumococcal vaccination recommendations at the time (PCV13-PPSV23 or PPSV23-PCV13, per ACIP recommendation [5]), showed that only 16.8% had received the complete vaccination, with 34.3% receiving only one dose (11.6% PPSV23, 22.7% PCV13) and 49% not receiving any pneumococcal vaccine, highlighting the challenges of sequential vaccination [15]. Moreover, during an outbreak of serotype 8 in an elderly population previously vaccinated with PPSV23, researchers found that the median number of years since PPSV was significantly higher for cases than non-cases, suggesting waning immunity [92]. Furthermore, in adults aged 65 years and above, PPSV23 showed a modest trend towards avoidance of CAP-related hospitalization and prevention of death or ICU admission in hospitalized CAP patients [93], whereas PCV13 ensured protection against disease by vaccinal serotypes [94].

Altogether, these data suggest that the higher-coverage PCVs (namely PCV20) pose advantages over the PCV15, PPSV23 and PCV15+PPSV23 schemes, and that improved vaccination strategies should be considered in the future.

### 4.4. Strengths and Limitations

Our study has some inherent limitations. Although we defined PCV eras in order to simplify the stratification of data (adjusting the results when the individual studies defined one of our PCV eras with different dates), not all European countries implemented PCV7 and PCV10/13 simultaneously, the rates of pediatric vaccination vary greatly between countries and even regions, and PCV10/13 only became widespread by late PCV10/13 era in some countries, all of which might shift the results in some regions considered. Moreover, although we grouped PCV10 and PCV13 in the same era, it is known that the choice of vaccine influenced the evolution of serotype prevalence [21]. Moreover, the epidemiology of pneumococcal disease has been changing rapidly, and thus data from the early epidemiological years of a certain time period are often quite different from data from the late years, which could account for some of the differences observed between countries. Furthermore, some of the studies only presented data for the whole population or for particular age groups (such as 5–64 years old), which we tried to mitigate by signalizing these occurrences in Supplementary Tables S2–S5 and mostly including studies in adults in our results sections. Additionally, differences in study size may have biased the importance of certain serotypes in a given PCV era. Another limitation is that geographical differences may exist in surveillance systems for IPD (countries with compulsory reporting and national coverage, countries with voluntary sentinel systems), while surveillance for non-invasive disease is limited and far more heterogeneous. Finally, it has to be noted that there may be heterogeneity among the different studies in their definition of the clinical outcomes, but data were reported as included in the relevant sources.

However, our study also has several strengths. It is an unbiased account of all the records from the past 10–12 years regarding the incidence, prevalence, severity, lethality, and antimicrobial resistance of all the additional PCV20 serotypes. It answers the need for an update on the changing epidemiology of invasive and non-invasive pneumococcal disease in Europe (which, as noted, presents an epidemiological profile different from that in the US [8]) and in adults/elderly (which represent a significant proportion of the pneumococcal infections). Our collected and summarized data are presented in Supplementary Tables S2–S5, becoming available to the scientific and medical community, and potentially serving as basis for future health policies regarding pneumococcal disease and vaccination in European adults.

## 5. Conclusions

In this systematic review, we found that the additional PCV20 serotypes have been emerging in adults across Europe over the past decade, representing a significant proportion of cases (8, 12F, 22F), more serious disease and/or lethality (10A, 11A, 15B, 22F), showing antimicrobial resistance (11A, 15B, 33F), and/or affecting more vulnerable individuals such as the elderly, immunocompromised patients, and patients with comorbidities (8, 10A, 11A, 15B, 22F). Importantly, out of these serotypes, only 22F and 33F are covered in the new PCV15, and PPSV23 has showed limitations in prevention of non-bacteremic pneumonia; therefore, PCV20 has the potential to address an unmet medical need. Altogether, our data suggest that older and/or more vulnerable patients (and adults in general) would benefit from vaccination with higher-coverage PCVs.

## Data Availability

If additional information in needed please contact the corresponding author.

## Supplementary Materials

The following supporting information can be downloaded at: https://www.mdpi.com/article/10.3390/microorganisms11061376/s1, Table S1: Search strings used to define the best search strategy for the PubMed and MEDLINE databases. Table S2: Summary of the findings from the pre-PCV7 era (pre-2001, unless stated otherwise by the authors of each paper). Table S3: Summary of the findings from the PCV7 era (2001-2009, unless stated otherwise by the authors of each paper). Table S4: Summary of the findings from the PCV10/13 era (2010-2015, unless stated otherwise by the authors of each paper).Table S5: Summary of the findings from the post-PCV10/13 era (2016-2022, unless stated otherwise by the authors of each paper). Figure S1: Number of records included in the study per country.
