# Supplementary material for: Pneumococcal Serotype Evolution and Burden in European Adults in the Last Decade: A Systematic Review
Source: Microorganisms. 2023 May 24;11(6):1376. doi: 10.3390/microorganisms11061376 (PMC10303156; doi:10.3390/microorganisms11061376)
Supplement: Supplementary file 1 [file microorganisms-11-01376-s001.zip › microorganisms-2305773-supplementary.pdf]

Supplementary Information

Table S1. Search strings used to define the best search strategy for the PubMed and MEDLINE databases.

Search	Search string	Records 2010-2022 [&]
Pneumococcal infections	Pneumococcal Infections[MeSH Major Topic]	6863
	Pneumococcal Infections[MeSH Major Topic] AND Europe[MeSH Terms] AND Adult[MeSH Terms]	534
	Pneumococcal Infections[MeSH Major Topic] AND Europe[MeSH Terms] AND (adult)	553
Streptococcus pneumoniae	Streptococcus Pneumoniae[MeSH Major Topic]	5687
	Streptococcus Pneumoniae[MeSH Major Topic] AND Europe[MeSH Terms] AND Adult[MeSH Terms]	267
	Streptococcus Pneumoniae[MeSH Major Topic] AND Europe[MeSH Terms] AND (adult)	279
Overlap check	*Streptococcus Pneumoniae[MeSH Major Topic] AND Europe[MeSH Terms] AND (adult) NOT Pneumococcal Infections[MeSH Major Topic]*	*36*
(Pneumococcal) serotype	Europe[MeSH Terms] AND (adult) AND (serotype)	1025
	Europe[MeSH Terms] AND (adult) AND (pneumococcal serotype)	341
	Pneumococcal Infections[MeSH Major Topic] AND (serotype)	2899
	Pneumococcal Infections[MeSH Major Topic] AND Europe[MeSH Terms] AND Adult[MeSH Terms] AND (serotype)	290
	**Pneumococcal Infections[MeSH Major Topic] AND Europe[MeSH Terms] AND (adult) AND (serotype)**	**304**
Overlap check	***Europe[MeSH Terms] AND (adult) AND (pneumococcal serotype) NOT Pneumococcal Infections[MeSH Major Topic]***	***37 ****
Serotype 8	Europe[MeSH Terms] AND (adult) AND (pneumococcal serotype 8)	157
Overlap check	***Europe[MeSH Terms] AND (adult) AND (pneumococcal serotype 8) NOT Pneumococcal Infections[MeSH Major Topic]***	***12 [$]***

Serotype 10A	Europe[MeSH Terms] AND (adult) AND (pneumococcal serotype 10A)	5
Overlap check	*Europe[MeSH Terms] AND (adult) AND (pneumococcal serotype 10A) NOT Pneumococcal Infections[MeSH Major Topic]*	*0 [$]*
Serotype 11A	Europe[MeSH Terms] AND (adult) AND (pneumococcal serotype 11A)	24
Overlap check	***Europe[MeSH Terms] AND (adult) AND (pneumococcal serotype 11A) NOT Pneumococcal Infections[MeSH Major Topic]***	***3 [$]***
Serotype 12F	Europe[MeSH Terms] AND (adult) AND (pneumococcal serotype 12F)	24
Overlap check	*Europe[MeSH Terms] AND (adult) AND (pneumococcal serotype 12F) NOT Pneumococcal Infections[MeSH Major Topic]*	*1 [$]* *(children)*
Serotype 15B	Europe[MeSH Terms] AND (adult) AND (pneumococcal serotype 15B)	8
Overlap check	*Europe[MeSH Terms] AND (adult) AND (pneumococcal serotype 15B) NOT Pneumococcal Infections[MeSH Major Topic]*	*0 [$]*
Serotype 22F	Europe[MeSH Terms] AND (adult) AND (pneumococcal serotype 22F)	28
Overlap check	*Europe[MeSH Terms] AND (adult) AND (pneumococcal serotype 22F) NOT Pneumococcal Infections[MeSH Major Topic]*	*1 [$]* *(unrelated)*
Serotype 33F	Europe[MeSH Terms] AND (adult) AND (pneumococcal serotype 33F)	10
Overlap check	*Europe[MeSH Terms] AND (adult) AND (pneumococcal serotype 33F) NOT Pneumococcal Infections[MeSH Major Topic]*	*0 [$]*

[&] Between the 1st of January 2010 and the 20th of April 2022 * Enough entries to justify conducting this search

[$] Serotype 8 and serotype 11A are the only "single serotype search" worth adding

Supplementary Table S2 - Summary of the findings from the pre-PCV7 era (pre-2001, unless stated otherwise by the authors of each paper).

	18-64yo (IPD)	≥65yo (IPD)	18-64yo (other)	≥65yo (other)	Severity and Lethality	Antimicrobial resistance
**8**	• 5.1% prevalence in Spain [1]*<br>• 3.1% pneumococcal meningitis isolates in Germany [3]<br>• 7% prevalence in Czech Republic [4]<br>• 6.6% prevalence in England [6]*<br>• Among the most common serotypes in Scotland (6%) [7]*[#]<br>• 6.5% prevalence in Ireland (2000-2001) – 5th most prevalent [9]*<br>• Among the most common serotypes in pneumococcal meningitis in the Netherlands (6%) [8]<br>• 7.8% prevalence in the Netherlands (2004-2006) [10][$]<br>• Mean prevalence in Denmark 0.63 [12][$]<br>• 3.5% prevalence in Sweden [11]<br>• 0.38 in 100.000 prevalence in Norway [14][§]	• 8.1% prevalence in the Netherlands (2004-2006) [10]<br>• Mean prevalence in Denmark 3.23 [12]			• Does not associate with greater risk of respiratory failure upon IPP (Spain) [2].<br>• Caused IPD more frequently in Spanish smokers (OR 3.28) [5]<br>• Does not associate with risk of hearing loss upon pneumococcal meningitis (Netherlands) [8].<br>• Significantly associated with a lower propensity for death OR 0.7 (individuals ≥5yo, IPD, Netherlands) [10][#]<br>• Increased risk of septic shock OR 2.37 [11]<br>• 6.6% of the isolates of pneumococcal empyema in Spain [13].	
**10A**	• 0.1% prevalence in Spain [1]*<br>• 3.6% pneumococcal meningitis isolates in Germany [3]<br>• 0.3% prevalence in England [6]*<br>• 1.5% prevalence in the Netherlands (2004-2006) [10][$]<br>• 0.18 in 100.000 prevalence in Norway [14][§]	• 1.5% prevalence in the Netherlands (2004-2006) [10]			• Significantly associated with a higher propensity for meningitis OR 3.7 (individuals ≥5yo, IPD, Netherlands) [10][#]	
**11A**	• 1.5% prevalence in Spain [1]*<br>• 2.1% pneumococcal meningitis isolates in Germany [3]<br>• 1.5% prevalence in England [6]*<br>• 0.7% prevalence in the Netherlands (2004-2006) [10][$]<br>• Mean prevalence in Denmark 0.16 [12][$]<br>• 0.25 in 100.000 prevalence in Norway [14][§]	• 1.4% prevalence in the Netherlands (2004-2006) [10]<br>• Mean prevalence in Denmark 1.03 [12][$]	• Among the most common serotypes in NIPP in Portugal (6.7%) [15][#]<br>• Significantly associated with NIPP vs IPD (Portugal) [15][#]		• Among the most common serotypes in patients with haematological malignancy in Finland (6.4%) [16]*<br>• Caused IPD more frequently in Spanish alcohol abusers (OR 3.28) [5]<br>• Significantly higher (OR 2.72) risk of mortality (IPD, Sweden) [17]*<br>• Significantly associated with a higher propensity for death OR 1.9 (IPD, Netherlands) [10][#§]	

Note: items spanning both IPD columns are listed under 18-64yo (IPD); the 11A "other" entries span both "other" columns.

	18-64yo (IPD)	≥65yo (IPD)	18-64yo (other)	≥65yo (other)	Severity and Lethality	Antimicrobial resistance
**12F**	• 2.9% prevalence in Spain [1]*				• Does not associate with greater risk of respiratory failure upon IPP (Spain) [2].	
	• 1.7% pneumococcal meningitis isolates in Germany [3]				• Significantly lower OR (0.29) for pneumonia (IPD, Sweden) [17]*	
	• 2.8% prevalence in England [6]*				• Significantly higher OR (3.03) for septicaemia (IPD, Sweden) [17]*	
	• 2.4% prevalence in the Netherlands (2004-2006) [10]$	• 1.2% prevalence in the Netherlands (2004-2006) [10]			• Significantly associated with hearing loss after pneumococcal meningitis in Denmark [18]*#	
	• Mean prevalence in Denmark 0.49 [12]$	• Mean prevalence in Denmark 2.65 [12]$				
	• Proportion of cases 12F increased, serotype more predominantly encountered in the adult population (Denmark) [19].					
	• 0.35 in 100.000 prevalence in Norway [14]§					
**15B**	• 0.6% prevalence in England [6]*					
	• 1.0% pneumococcal meningitis isolates in Germany [3].					
	• 0.9% prevalence in the Netherlands (2004-2006) [10]$					
	• 0.13 in 100.000 prevalence in Norway [14]§&					
**22F**	• 1.4% prevalence in Spain [1]*		• Among the most common serotypes in NIPP in Portugal (4.1%) [15]#		• Does not associate with risk of hearing loss upon pneumococcal meningitis (Netherlands) [8]	
	• 3.5% pneumococcal meningitis isolates in Germany [3]				• Significantly lower OR (0.40) for pneumonia (IPD, Sweden) [17]*	
	• 2.1% prevalence in England [6]*				• Significantly higher OR (3.13) for septicaemia (IPD, Sweden) [17]*	
	• Among the most common serotypes in pneumococcal meningitis in the Netherlands (5%) [8]				• Significantly associated with a lower propensity for empyema OR 0.3 (individuals ≥5yo, IPD, Netherlands) [10]#	
	• 2.6% prevalence in the Netherlands (2004-2006) [10]$	• 1.7% prevalence in the Netherlands (2004-2006) [10]			• Significantly associated with a higher propensity for meningitis OR 2.0 (individuals ≥5yo, IPD, Netherlands) [10]#	
	• Mean prevalence in Denmark 0.36 [12]$	• Mean prevalence in Denmark 2.23 [12]$				
	• 0.86 in 100.000 prevalence in Norway [14]§					
**33F**	• 1.3% prevalence in England [6]*					
	• 1.7% pneumococcal meningitis isolates in Germany [3].					

	18-64yo (IPD)	≥65yo (IPD)	18-64yo (other)	≥65yo (other)	Severity and Lethality	Antimicrobial resistance
	• 2.2% prevalence in the Netherlands (2004-2006) [10][$]	• 0.8% prevalence in the Netherlands (2004-2006) [10]				
	• Mean prevalence in Denmark 0.11 [12][$]	• Mean prevalence in Denmark 1.04 [12][$]				
	• 0.33 in 100.000 prevalence in Norway [14][§]					

* total population, [$] 5-64 years, [§] ≥ 5 years, [&] 15B/C, [#] cumulative prevalence with other PCV eras (PCV7 or PCV7 and PCV10/13)

(N)IPP (non-)invasive pneumococcal pneumonia, OR odds ratio

References

1. Fenoll, A., Granizo, J. J., Giménez, M. J., Yuste, J. & Aguilar, L. Secular trends (1990-2013) in serotypes and associated non-susceptibility of S. pneumoniae isolates causing invasive disease in the pre-/post-era of pneumococcal conjugate vaccines in Spanish regions without universal paediatric pneumococcal vaccination. *Vaccine* **33**, 5691–5699 (2015).

2. Burgos, J. *et al.* Risk factors for respiratory failure in pneumococcal pneumonia: The importance of pneumococcal serotypes. *European Respiratory Journal* **43**, 545–553 (2014).

3. Imöhl, M. *et al.* Pneumococcal meningitis and vaccine effects in the era of conjugate vaccination: Results of 20 years of nationwide surveillance in Germany. *BMC Infectious Diseases* **15**, (2015).

4. Žemličková, H. *et al.* Clonal distribution of invasive pneumococci, Czech Republic, 1996-2003. *Emerging Infectious Diseases* **16**, 287–289 (2010).

5. Grau, I. *et al.* Smoking and alcohol abuse are the most preventable risk factors for invasive pneumonia and other pneumococcal infections. *International Journal of Infectious Diseases* **25**, 59–64 (2014).

6. Moore, C. E. *et al.* Reduction of invasive pneumococcal disease 3 years after the introduction of the 13-valent conjugate vaccine in the Oxfordshire region of England. *Journal of Infectious Diseases* **210**, 1001–1011 (2014).

7. Lamb, K. E. *et al.* Trends in serotypes and sequence types among cases of invasive pneumococcal disease in Scotland, 1999-2010. *Vaccine* **32**, 4356–4363 (2014).

8. Heckenberg, S. G. B., Brouwer, M. C., van der Ende, A., Hensen, E. F. & van de Beek, D. Hearing loss in adults surviving pneumococcal meningitis is associated with otitis and pneumococcal serotype. *Clinical Microbiology and Infection* **18**, 849–855 (2012).

9. Vickers, I. *et al.* Serotype distribution of Streptococcus pneumoniae causing invasive disease in the Republic of Ireland. *Epidemiology and Infection* **139**, 783–790 (2011).

10. Wagenvoort, G. H. J. *et al.* Invasive pneumococcal disease: Clinical outcomes and patient characteristics 2–6 years after introduction of 7-valent pneumococcal conjugate vaccine compared to the pre-vaccine period, the Netherlands. *Vaccine* **34**, 1077–1085 (2016).

11. Ahl, J. *et al.* High prevalence of septic shock caused by Streptococcus pneumoniae serotype 3 - a retrospective epidemiological study. *BMC Infectious Diseases* **13**, (2013).

12. Slotved, H. C., Dalby, T. & Hoffmann, S. The effect of pneumococcal conjugate vaccines on the prevalence of invasive pneumococcal disease caused by ten non-vaccine serotypes in Denmark. *Vaccine* **34**, 769–774 (2016).

13. Burgos, J. *et al.* The spectrum of pneumococcal empyema in adults in the early 21st century. *Clinical Infectious Diseases* **53**, 254–261 (2011).

14. Vestrheim, D. F. *et al.* Indirect effect of conjugate pneumococcal vaccination in a 2 + 1 dose schedule. *Vaccine* **28**, 2214–2221 (2010).

15. Horácio, A. N., Lopes, J. P., Ramirez, M. & Melo-Cristino, J. Non-invasive pneumococcal pneumonia in Portugal - Serotype distribution and antimicrobial resistance. *PLoS ONE* **9**, (2014).

16. Lindström, V. *et al.* Invasive pneumococcal disease in patients with haematological malignancies before routine use of conjugate vaccines in Finland. *Infectious Diseases* **48**, 399–402 (2016).

17. Browall, S. *et al.* Clinical manifestations of invasive pneumococcal disease by vaccine and non-vaccine types. *European Respiratory Journal* **44**, 1646–1657 (2014).

18. Worsøe, L., Cayé-Thomasen, P., Brandt, C. T., Thomsen, J. & Østergaard, C. Factors associated with the occurrence of hearing loss after pneumococcal meningitis. *Clinical Infectious Diseases* **51**, 917–924 (2010).

19. Harboe, Z. B. *et al.* Temporal trends in invasive pneumococcal disease and pneumococcal serotypes over 7 decades. *Clinical Infectious Diseases* **50**, 329–337 (2010).

Supplementary Table S3 - Summary of the findings from the PCV7 era (2001-2009, unless stated otherwise by the authors of each paper).

	18-64yo (IPD)	≥65yo (IPD)	18-64yo (other)	≥65yo (other)	Severity and Lethality	Antimicrobial resistance
**8**	• 3.2% prevalence in Spain [1]*		• 3.1% prevalence of uncomplicated CAP in Spain [2]		• Does not associate with greater risk of respiratory failure upon IPP (Spain) [3].	• Among the most frequent serotypes showing ERY resistance 29.4% (only in the PCV7 era, IPD, Spain) [4]
	• 4.5% prevalence in bacteremic pneumococcal community-acquired pneumonia (BPP) in Spain [5]		• 6% prevalence in CAP in Spain [6]*		• 1.7% of the isolates of pneumococcal empyema in Spain [7].	
	• 0.35 per 100.000 prevalence in Catalonia, Spain [8]*					
	• 0.73 in 100.000 prevalence in Spain [4]		• 1.3% prevalence NIPD in Greece [9]		• Caused IPD more frequently in Spanish smokers (OR 3.28) [10]	• 61.8% of the serotype 8 isolates were co-resistant to ERY, LVX and tetracycline (IPD, Spain) [11]*
	• 13.5% prevalence in Spain [11]*					
	• Main serotype causing recurrent IPD in Spain (12.1%) [12]*#				• Does not associate with risk of hearing loss upon pneumococcal meningitis (Netherlands) [13].	
	• 5.2% prevalence in Spain [14]	• 5.8% prevalence in Spain [14]				• Serotype 8 represented 14% of the NFX- and LVX-resistant isolates (MIC > 256 ug/mL and > 1.5-2 ug/mL) (IPD, Spain) [16]*
		• 5.0% prevalence in individuals aged 60 and up, Madrid, Spain [15]#				
		• 4-5% prevalence in bacteremic pneumonia and 14-21% prevalence in primary bacteremia in South Catalonia, Spain (2002-2009) [17]			• Significantly associated with lower CFR (IPD, Netherlands) [18]*	
	• Significantly decreased vs pre-PCV7 era in Portugal [19]				• Significantly less common in high-risk group patients (IPD, Netherlands) [20]#	
	• Among the most common serotypes in Portugal (6.2%) [21]#					
	• 5.7% prevalence in Portugal - 5th most prevalent [22]				• Significantly associated with a lower propensity for death OR 0.7 (individuals ≥5yo, IPD, Netherlands) [23]	• Serotype 8 accounted for 0.6% of the PNSP (IPD, Denmark) [24]
	• Significantly more frequent in the younger adults (18-49) 10.5% (Portugal) [21]#	• vs 4.5% in ≥65yo [21]#			• Detected in 7.5% of CAP hospitalized patients with pneumococcal bacteraemia (UK) [25]	
	• 9.1% prevalence in 18-49yo, 4.5% in 50-64yo in Portugal [26]#	• 4.2% prevalence in Portugal [26]#				
	• 1% prevalence in pneumococcal meningitis in France (2008-2009) [27]*				• Serotype 8 was significantly more frequent among HIV-infected patients (36.5%) (IPD, Spain) [11]*	
		• 2.6%, 1.6%, 2.1% prevalence in France (2001/02, 2003/05, 2006/10) [28]				
	• 3.5% prevalence in Italy (2009) [29]*				• Serotype 8 was found to have enhanced propensity to cause invasive disease OR 46.2 [22]	
	• 3.9% prevalence in Italy [30]					
	• 2.1% pneumococcal meningitis isolates in Germany [31].					
	• 1.5% prevalence in Poland (2009) [32]*					

	18-64yo (IPD)	≥65yo (IPD)	18-64yo (other)	≥65yo (other)	Severity and Lethality	Antimicrobial resistance
	• 5.0% (15-24yo) and 3.6% (25-54yo) prevalence in Poland [34]	• 1.9% prevalence in Poland [34]			• Among the serotypes with lowest CFR (6%), and the lowest QALY loss (1.17) (IPD, England) [33]$	
	• 18.9% prevalence in England [35]*					
	• 0.58 per 100.000 prevalence in England, but not detected in carriage [36]*					
	• 7.5% prevalence in North East England [37]*#					
	• 0.5-0.8 in 100.000 prevalence in IPD in North East England (2006-2009) [38]*					
	• 3rd most prevalent serotype in invasive and non-invasive pneumococcal CAP in the UK, 9.6% prevalence [25]					
	• 7.3% prevalence of pneumococcal meningitis in England and Wales (2004-2009) [39]					
	• Among the most prevalent serotypes in England and Wales (2009-2010) [40]$					
	• Significant decrease in prevalence vs pre-PCV7 era in England and Wales IRR 0.58 (2008-2010) [40]$					
	• Among the most common serotypes in Scotland (6%) [41]*					
	• Significant decrease in prevalence vs pre-PCV7 era in Ireland (2007-2008) [42]*					
		• 0.64 per 100.000 prevalence in Ireland (2007-2008) [43]				
	• Among the most common serotypes in pneumococcal meningitis in the Netherlands (6%) [13]					
	• 8% prevalence in community acquired bacterial (pneumococcal) meningitis in the Netherlands [44]					
	• Third most prevalent serotype (2008-2010) in the Netherlands [45]*					
	•	• 12% prevalence in the Netherlands [46]#				
	• 10.5% prevalence in the Netherlands (2004-2006) [23]$	• 10.8% prevalence in the Netherlands (2004-2006) [23]				
		• Significantly increasing vs pre-PCV7 era in IPD in Denmark for 85+ yo (IRR 1.97) [47]				
	• Mean prevalence in Denmark 0.62 [48]$	• Mean prevalence in Denmark 3.84 [48]$				
		• Among the most predominant serotypes in Denmark, particularly in this population [49]				
	• 0.42 per 100.000 prevalence in Sweden (2009) [50]*					
	• 3.4% prevalence of pneumonia, 3.5% prevalence of meningitis and 1.4% prevalence of septicaemia in Norway (2008) [51]*					

	18-64yo (IPD)	≥65yo (IPD)	18-64yo (other)	≥65yo (other)	Severity and Lethality	Antimicrobial resistance
	• 0.48 in 100.000 prevalence in Norway – increased vs pre-PCV7 era IRR 1.25 [52]§					
**10A**	• 0.8% prevalence in Spain [1]*		• 3.5% prevalence in NIPD in Spain – increased vs pre-PCV7 era (1.8%) [53]*		• High CFP in meningitis in Poland 22% [32]*	• Serotype 10A accounted for 0.6% of the PNSP (IPD, Denmark) [24]
	• 0.30 per 100.000 prevalence in Catalonia, Spain [8]*				• Significantly more common in high-risk group patients 39% vs 16% (average) – significantly associated with IPD in high-risk patients (Netherlands) [20]#	
	• 0.18 in 100.000 prevalence in Spain [4]					
		• 14-21% prevalence in primary bacteremia in South Catalonia, Spain (2002-2009) [17]	• 1.9% prevalence of complicated CAP in Spain [2]			
		• 1.6%, 0.9%, 1.0% prevalence in France (2001/02, 2003/05, 2006/10) [28]	• 3% prevalence in CAP in Portugal [6]*<br>• 1.9% prevalence NIPD in Greece [9]		• Significantly associated with a higher propensity for meningitis OR 3.7 (individuals ≥5yo, IPD, Netherlands) [23]#	
	• 2% prevalence in pneumococcal meningitis in France (2008-2009) [27]*					
	• 2.2% prevalence in Italy (2009) [29]*				• Very associated with meningitis (IPD, Norway) [51]*	
	• 5.0% pneumococcal meningitis isolates in Germany [31].					
	• 3.0% prevalence in Poland (2009) [32]*				• More associated with meningitis and septicemia than with pneumonia (IPD, Norway) [51]*	
	• 4.1% (25-54yo) and 2.1% (55-64) prevalence in Poland [34]	• 1.9% prevalence in Poland [34]				
	• 1.0% prevalence in England [35]*				• Significantly more likely to cause meningitis (isolated in 32% of the isolates) (IPD, England) [33]$	
	• 1.2% prevalence in North East England [37]*#					
	• 1.3% prevalence of pneumococcal meningitis in England and Wales (2004-2009) [39]					
	• 2.2% prevalence in the Netherlands (2004-2006) [23]$	• 1.8% prevalence in the Netherlands (2004-2006) [23]				
	• 0.13 per 100.000 prevalence in Sweden (2009) [50]*					
	• 0.7% prevalence of pneumonia, 40% prevalence of meningitis and 2.5% prevalence of septicaemia in Norway (2009) [51]*					
	• 0.32 in 100.000 prevalence in Norway – increased vs pre-PCV7 era IRR 1.73 [52]§					
**11A**	• 1.8% prevalence in Spain [1]*		• Among the most common serotypes in NIPP in Portugal (6.7%) [54]		• Caused IPD more frequently in Spanish alcohol abusers (OR 3.28) [10]	• 5.3% of serotype 11A isolates showed resistance to PEN and CTX (MIC > 0.06 mg/L and > 0.5 mg/L) (IPD, Poland) [34]*
	• 0.24 per 100.000 prevalence in Spain [55]				• Significantly higher (OR 2.72) risk of mortality (IPD, Sweden) [56]*	
	• 0.23 in 100.000 prevalence in Spain [4]		• Significantly associated with NIPP vs IPD (Portugal) [54]			
	• 0.10 per 100.000 prevalence in Catalonia, Spain [8]*				• Significantly associated with higher CFR (IPD, Netherlands) [18]*	• Serotype 11A represented 14.3% of the isolates resistant to ERY (IPD, Spain) [57]
		• 3.8% prevalence in individuals aged 60 and up, Madrid, Spain [15]#	• 5.3% prevalence in NIPD in Spain – emerging vs pre-PCV7 era [53]*			

	18-64yo (IPD)	≥65yo (IPD)	18-64yo (other)	≥65yo (other)	Severity and Lethality	Antimicrobial resistance
		• 2-2.5% prevalence in bacteremic pneumonia in South Catalonia, Spain (2002-2009) [17]	• Most frequently carried serotype in the parents of vaccinated children in the Netherlands (2%) [59]		• IPD patients with serotype 11A were significantly older than those infected with other vaccinal serotypes (Sweden) [58]*	• Serotype 11A accounted for 1.7% of the PNSP (IPD, Denmark) [24]
	• 2.8% prevalence in 18-49yo, 3.4% in 50-64yo in Portugal [26]#	• 2.5% prevalence in Portugal [26]#			• Significantly associated with a higher propensity for death OR 1.9 (individuals ≥5yo, IPD, Netherlands) [23]	
	• 2.6% prevalence in Italy (2009) [29]*		• 3.9% prevalence NIPD in Greece [9]			• Non-susceptibility rates of around 35% for PEN, AMOX, CXM and ERY (NIPD, Spain) [53]*
	• 2.4% prevalence in Greece [9]				• Serotype 11A was associated with carriage, having a lower invasive disease potential (Portugal) OR 0.37 [22]	
	• 2.1% pneumococcal meningitis isolates in Germany [31].					
	• 3.0% prevalence in Poland (2009) [32]*					
	• 5.0% (15-24yo), 3.6% (25-54yo) and 1.0% (55-64) prevalence in Poland [34]	• 2.5% prevalence in Poland [34]			• Among the serotypes with highest CFR (30%), and the highest QALY loss (5.82) (IPD, England) [33]$	
	• 5.3% prevalence in Hungary [60]*					
	• 7.5% prevalence in England [35]*					
	• 1.7% prevalence in North East England [37]*#					
	• 0.1-0.2 in 100.000 prevalence in IPD in North East England (2006-2009) [38]*					
	• 1.2% prevalence of pneumococcal meningitis in England and Wales (2004-2009) [39]					
	• Responsible for at least 1% of IPD in Scotland (2006-2009) [41]*					
		• 0.64 per 100.000 prevalence in Ireland (2007-2008) [43]				
	• Mean prevalence in Denmark 0.19 [48]$	• Mean prevalence in Denmark 1.67 [48]$				
		• 1.7% prevalence in the Netherlands [46]#				
	• 1.6% prevalence in the Netherlands (2004-2006) [23]$	• 1.8% prevalence in the Netherlands (2004-2006) [23]				
	• 0.26 per 100.000 prevalence in Sweden (2009) [50]*					
	• 4.0% prevalence in acute otitis media in Sweden [61]*					
	• 2.4% prevalence of pneumonia, 5.3% prevalence of meningitis in Norway (2008) [51]*					
	• 0.39 in 100.000 prevalence in Norway – increased vs pre-PCV7 era IRR 1.55 [52]$					
	• 4.9 per 100.000 prevalence in Iceland [62]	• 26.4 per 100.000 prevalence in Iceland [62]				
**12F**	• 2.3% prevalence in Spain [1]*		• 5.7% prevalence of complicated CAP in Spain [2]		• Does not associate with greater risk of respiratory failure upon IPP (Spain) [3]	• Serotype 12F represented 7.1% of the isolates resistant to PEN and 7.1% of the
	• 0.34 in 100.000 prevalence in Spain [4]					
	• 4.0% prevalence in Spain [11]*		• 5% prevalence in CAP in the UK [6]*			
	• 0.55 per 100.000 prevalence in Catalonia, Spain [8]*					

	18-64yo (IPD)	≥65yo (IPD)	18-64yo (other)	≥65yo (other)	Severity and Lethality	Antimicrobial resistance
	• 2.8% prevalence in Spain [14]	• 3.1% prevalence in Spain [14]			• Significant decrease in prevalence vs pre-PCV7 era in England and Wales IRR 0.73 (2008-2010) [40]$	isolates resistant to ERY (IPD, Spain) [57]
		• 3.1-3.8% prevalence in bacteremic pneumonia in South Catalonia, Spain (2002-2009) [17]			• Significantly lower OR (0.29) for pneumonia (IPD, Sweden) [56]*	
	• 3% prevalence in pneumococcal meningitis in France (2008-2009) [27]*				• Significantly higher OR (3.03) for septicaemia (IPD, Sweden) [56]*	
	• 4.7% prevalence in France, 7th most prevalent serotype [63]				• Significantly associated with hearing loss after pneumococcal meningitis in Denmark [64]*	
		• 0.2%, 0.3%, 2.1% prevalence in France (2001/02, 2003/05, 2006/10) [28]				
	• 2.2% prevalence in Italy (2009) [29]*				• Significantly associated with lower CFR (IPD, Netherlands) [18]*	
	• 8% prevalence in Belgium, 5th most prevalent serotype [65]#					
	• 2.7% pneumococcal meningitis isolates in Germany [31].				• Significantly less common in high-risk group patients (IPD, Netherlands) [20]#	
	• 2.7% prevalence in Poland (2008) [32]*					
	• Significantly more common in persons aged above 25 years (89% occurred in adults, IPD, Poland) [34]				• CFR 6% (IPD, Belgium) [65]#	
	• 12F: 5.0% (15-24yo), 4.1% (25-54yo) and 9.3% (55-64) prevalence in Poland [34]	• 8.6% prevalence in Poland [34]				
	• 5.9% prevalence in England [35]*					
	• 0.25 per 100.000 prevalence in England, but not detected in carriage [36]*					
	• 2.9% prevalence in North East England [37]*#					
	• 0.2-0.3 in 100.000 prevalence in IPD in North East England (2006-2009) [38]*					
	• 4.7% prevalence of pneumococcal meningitis in England and Wales (2004-2009) [39]					
	• Significant decrease in prevalence vs pre-PCV7 era in England and Wales IRR 0.73 (2008-2010) [40]$					
	• Responsible for at least 1% of IPD in Scotland (2006-2009) [41]*					
		• 1.06 per 100.000 prevalence in Ireland (2007-2008) [43]				
		• 2.6% prevalence in the Netherlands [46]#				
	• 2.2% prevalence in the Netherlands (2004-2006) [23]$	• 3.4% prevalence in the Netherlands (2004-2006) [23]				
	• Mean prevalence in Denmark 0.45 [48]$	• Mean prevalence in Denmark 2.13 [48]$				

	18-64yo (IPD)	≥65yo (IPD)	18-64yo (other)	≥65yo (other)	Severity and Lethality	Antimicrobial resistance
	• 0.09 per 100.000 prevalence in Sweden (2009) [50]*					
	• 0.5% prevalence of pneumonia in Norway (2008) [51]*					
	• 0.14 in 100.000 prevalence in Norway – decreased vs pre-PCV7 era IRR 0.38 [52]§					
**15B**	• 0.07 in 100.000 prevalence in Spain [4]		• Among the most prevalent serotypes in pneumococcal ocular infections in Spain 4.9% (increasing from 2.4% to 8.9% over time) [66]*		• Significantly associated with higher CFR (IPD, Netherlands) [18]*	• 20% of serotype 15B/C isolates showed resistance to PEN (MIC > 0.06 mg/L, IPD, Poland) [34]*
	• 0.13 per 100.000 prevalence in Catalonia, Spain [8]*				• Significantly more common in high-risk group patients 52% vs 16% (average) – significantly associated with IPD in high-risk patients (Netherlands) [20]#	• Serotype 15B accounted for 2.3% of the PNSP (IPD, Denmark) [24]
		• 1% prevalence in bacteremic pneumonia in South Catalonia, Spain (2002-2009) [17]			• More associated with meningitis and septicemia than with pneumonia (IPD, Norway) [51]*	
	• 2% prevalence in pneumococcal meningitis in France (2008-2009) [27]*				• Serotype 15B/C was associated with carriage, having a lower invasive disease potential (Portugal) OR 0.09 [22]	
		• 0.5%, 1.3%, 0.9% prevalence in France (2001/02, 2003/05, 2006/10) [28]&			• Significantly more likely to cause meningitis (isolated in 22% of the isolates) (IPD, England) [33]$	
	• 3.1% prevalence in Italy (2009) [29]*					
	• Significantly increasing vs pre-PCV7 era in IPD in Germany (1,2% vs 0.6%) [67,68]					
	• 3.1% pneumococcal meningitis isolates in Germany [31]					
	• 3.0% prevalence in Poland (2009) [32]*&					
	• 1.8% prevalence in England [35]*					
	• 1.2% prevalence of pneumococcal meningitis in England and Wales (2004-2009) [39]					
		• Significant increase in prevalence vs pre-PCV7 era in England and Wales IRR 2.23 (2008-2010) [40]				
	• Responsible for at least 1% of IPD in Scotland (2006-2009) [41]*					
	• 1.0% prevalence in the Netherlands (2004-2006) [23]$	• 1.2% prevalence in the Netherlands (2004-2006) [23]				
	• 0.09 per 100.000 prevalence in Sweden (2009) [50]*					
	• 2.1% prevalence in acute otitis media in Sweden [61]*					
	• 1% prevalence of pneumonia, 3.5% prevalence of meningitis and 2.7% prevalence of septicaemia in Norway (2008) [51]*					
	• 0.29 in 100.000 prevalence in Norway – increased vs pre-PCV7 era IRR 2.25 [52]§&					
	• 2.8 per 100.000 prevalence in Iceland [62]&	• 15.1 per 100.000 prevalence in Iceland [62]&				
**22F**	• 2.0% prevalence in Spain [1]*		• Among the most common serotypes in NIPP in Portugal (4.1%) [54]		• Does not associate with risk of hearing loss upon pneumococcal meningitis (Netherlands) [13]	
	• 4.5% prevalence in BPP in Spain [5]					
	• 0.34 in 100.000 prevalence in Spain [4]					

	18-64yo (IPD)	≥65yo (IPD)	18-64yo (other)	≥65yo (other)	Severity and Lethality	Antimicrobial resistance
	• 2% prevalence in Spain [14]	• 2.2% prevalence in Spain [14]	• 3.1% prevalence of uncomplicated CAP in Spain [2]		• Significantly lower OR (0.40) for pneumonia (IPD, Sweden) [56]*	
		• 3.6% prevalence in Spain [69]			• Significantly higher OR (3.13) for septicaemia (IPD, Sweden) [56]*	
	• 0.10 per 100.000 prevalence in Catalonia, Spain [8]*		• 7% prevalence in CAP in Belgium, 8% in the UK, 6% in Portugal, 3% in Spain, 5% in Denmark [6]*			
		• 1% prevalence in bacteremic pneumonia in South Catalonia, Spain (2002-2009) [17]			• High CFP in meningitis in Poland 29% [32]*	
		• 4.9% prevalence in individuals aged 60 and up, Madrid, Spain [15]#	• 1.3% prevalence NIPD in Greece [9]		• Percentage of septic shock was significantly higher in patients with serotype 22F in BPP [5]	
	• 10.7% prevalence in pneumococcal pneumonia (invasive plus non-invasive) in individuals aged 60 and up, in Southern Catalonia, Spain [70]				• Significantly associated with a lower propensity for empyema OR 0.3 (individuals ≥5yo, IPD, Netherlands) [23]	
	• 3.2% prevalence in 18-49yo, 3.4% in 50-64yo in Portugal [26]#	• 4.6% prevalence in Portugal [26]#			• Significantly associated with a higher propensity for meningitis OR 2.0 (individuals ≥5yo, IPD, Netherlands) [23]	
	• 4% prevalence in pneumococcal meningitis in France (2008-2009) [27]*					
		• 1.9%, 3.5%, 4.7% prevalence in France (2001/02, 2003/05, 2006/10) [28]			• CFR 12% (IPD, Belgium) [65]#	
	• 6.1% prevalence in Italy (2009) [29]*				• Detected in 7.5% of CAP hospitalized patients with pneumococcal bacteraemia (UK) [25]	
	• 5% prevalence in Belgium, 6th most prevalent serotype [65]#					
	• 6.3% pneumococcal meningitis isolates in Germany [31]					
	• Significantly increasing vs pre-PCV7 era in IPD in Germany (4.7% vs 2.3%) [68].				• Very associated with septicemia (IPD, Norway) [51]*	
	• 1.5% prevalence in Poland (2009) [32]*					
	• 2.3% (25-54yo) and 4.1% (55-64) prevalence in Poland [34]	• 2.5% prevalence in Poland [34]				
	• 0.24 per 100.000 prevalence in Austria (rank 8) [71]					
	• 20.7% prevalence in England [35]*					
	• Significant increase in England after vaccine implementation (IRR ~1.9) [72]*					
	• 6.8% prevalence in North East England [37]*#					
	• 0.1-0.9 in 100.000 prevalence in IPD in North East England (2006-2009) [38]*					
	• Significant increase in prevalence over time by 1192% (IPD, North East England) [38]$	• Significant increase in prevalence over time by 681% (IPD, North East England) [38]				

	18-64yo (IPD)	≥65yo (IPD)	18-64yo (other)	≥65yo (other)	Severity and Lethality	Antimicrobial resistance
	• 4.5% prevalence of pneumococcal meningitis in England and Wales (2004-2009) [39]					
	• Significant increase in prevalence vs pre-PCV7 era in England and Wales IRR 1.85 (2008-2010) [40]$	• Significant increase in prevalence vs pre-PCV7 era in England and Wales IRR 2.09 (2008-2010) [40]				
	• Significantly increasing vs pre-PCV7 era in Scotland (OR 1.34) [41]*					
		• 0.85 per 100.000 prevalence in Ireland (2007-2008) [43]				
	• Among the most common serotypes in pneumococcal meningitis in the Netherlands (5%) [13]					
	• 7% prevalence in community acquired bacterial (pneumococcal) meningitis in the Netherlands [44]#					
	• Significant increase in prevalence vs pre-PCV7 era in the Netherlands [45]*					
	• Significantly increased vs pre-PCV7 period in the Netherlands [73]*					
		• 9.4% prevalence in the Netherlands [46]#				
	• 4.6% prevalence in the Netherlands (2004-2006) [23]$	• 6.5% prevalence in the Netherlands (2004-2006) [23]				
		• Significant increase in prevalence vs pre-PCV7 era in the Netherlands in individuals aged 50 and up [74]				
	• Mean prevalence in Denmark 0.36 [48]$	• Mean prevalence in Denmark 3.22 [48]$				
		• Among the most predominant serotypes in Denmark, particularly in this population [49]				
	• Significantly increased vs pre-PCV7 era RR 3.29 (Sweden) [58]*					
	• 1.14 per 100.000 prevalence in Sweden (2009) [50]*					
	• 10.6% prevalence of pneumonia, 5.7% prevalence of meningitis and 25% prevalence of septicaemia in Norway (2009) [51]*					
	• 1.07 in 100.000 prevalence in Norway – increased vs pre-PCV7 era IRR 1.23 [52]§					
	• 5.6 per 100.000 prevalence in Iceland [62]	• 15.1 per 100.000 prevalence in Iceland [62]				
	• 4% of the IPD isolates in Finland (2009) [75]*					
**33F**	• Increase from the pre-PCV7 era in Spain [76]				• Significantly more common in high-risk group patients 30% vs 16% (average) –	• Increase in IPD prevalence from the pre-PCV7 era in Spain accompanied by
	• 0.2 in 100.000 prevalence in Spain [4]					
	• 0.08 per 100.000 prevalence in Catalonia, Spain [8]*					

	18-64yo (IPD)	≥65yo (IPD)	18-64yo (other)	≥65yo (other)	Severity and Lethality	Antimicrobial resistance
		• 7-9% prevalence in meningitis in South Catalonia, Spain (2002-2009) [17]			significantly associated with IPD in high-risk patients (Netherlands) [20]#	increased in macrolide resistant isolates [76].
	• 2% prevalence in pneumococcal meningitis in France (2008-2009) [27]*				• Detected in 7.5% of CAP hospitalized patients with pneumococcal bacteraemia (UK) [25]	• Emerged in Norway as a frequent serotype displaying resistance to several antimicrobials in IPD (ERY, SXT, CLI) [77]*
	• 1.3% prevalence in Italy (2009) [29]*					
	• 1.9% pneumococcal meningitis isolates in Germany [31]					
	• 7% prevalence in England [35]*					
	• 2.6% prevalence in North East England [37]*#					
	• 0.2-0.3 in 100.000 prevalence in IPD in North East England (2006-2009) [38]*					• Among the most frequent serotypes showing ERY resistance 12.9% (IPD, Spain) [4]
	• Significant increase in prevalence vs pre-PCV7 era in England and Wales IRR 1.58 (2008-2010) [40]$	• Significant increase in prevalence vs pre-PCV7 era in England and Wales IRR 2.04 (2008-2010) [40]				
	• 1.8% prevalence of pneumococcal meningitis in England and Wales (2004-2009) [39]					• Serotype 33F accounted for 0.6% of the PNSP (IPD, Denmark) [24]
	• Responsible for at least 1% of IPD in Scotland (2006-2009) [41]*					
		• 1.27 per 100.000 prevalence in Ireland (2007-2008) [43]				
		• 2.6% prevalence in the Netherlands [46]#				
	• 2.0% prevalence in the Netherlands (2004-2006) [23]$	• 2.3% prevalence in the Netherlands (2004-2006) [23]				
	• Mean prevalence in Denmark 0.18 [48]$	• Mean prevalence in Denmark 1.72 [48]$				
	• 0.24 per 100.000 prevalence in Sweden (2009) [50]*					
	• Significantly increased vs pre-PCV7 era RR 2.02 (Sweden) [58]*					
	• 3.6% prevalence of pneumonia and 5.0% prevalence of septicaemia in Norway (2009) [51]*					
	• 0.66 in 100.000 prevalence in Norway – increased vs pre-PCV7 era IRR 2.00 [52]§					
		• 15.1 per 100.000 prevalence in Iceland [62]				

* total population, $ 5-64 years, § ≥ 5 years, & 15B/C, # cumulative prevalence with other PCV eras (PCV10/13)

AMOX amoxicillin, BPP bacteremic pneumococcal community-acquired pneumonia, CFP case fatality proportion, CFR case fatality ratio, CLI clindamycin, CTX cefotaxime, CXM cefuroxime, ERY erythromycin, IRR prevalence rate ratio, LVX levofloxacin, MIC minimal inhibitory concentration, NFX norfloxacin, (N)IPP (non-)invasive pneumococcal pneumonia, OR odds ratio, PEN penicillin, SXT trimethoprim/sulfamethoxazole

References

1. Fenoll, A., Granizo, J. J., Giménez, M. J., Yuste, J. & Aguilar, L. Secular trends (1990-2013) in serotypes and associated non-susceptibility of S. pneumoniae isolates causing invasive disease in the pre-/post-era of pneumococcal conjugate vaccines in Spanish regions without universal paediatric pneumococcal vaccination. *Vaccine* **33**, 5691–5699 (2015).
2. Cillóniz, C. *et al.* Pulmonary complications of pneumococcal community-acquired pneumonia: prevalence, predictors, and outcomes. *Clinical Microbiology and Infection* **18**, 1134–1142 (2012).
3. Burgos, J. *et al.* Risk factors for respiratory failure in pneumococcal pneumonia: The importance of pneumococcal serotypes. *European Respiratory Journal* **43**, 545–553 (2014).
4. Càmara, J. *et al.* Decrease of invasive pneumococcal disease (IPD) in adults after introduction of pneumococcal 13-valent conjugate vaccine in Spain. *PLoS One* **12**, (2017).
5. España, P. P. *et al.* Evolution of serotypes in bacteremic pneumococcal adult pneumonia in the period 2001–2014, after introduction of the pneumococcal conjugate vaccine in bizkaia (spain). *Vaccine* **37**, 3840–3848 (2019).
6. Morrissey, I. & Leakey, A. Activity of ceftaroline against serotyped Streptococcus pneumoniae isolates from Europe and South Africa associated with community-acquired bacterial pneumonia (2007-08). *Journal of Antimicrobial Chemotherapy* **67**, 1408–1412 (2012).
7. Burgos, J. *et al.* The spectrum of pneumococcal empyema in adults in the early 21st century. *Clinical Infectious Diseases* **53**, 254–261 (2011).
8. Ciruela, P. *et al.* The changing epidemiology of invasive pneumococcal disease after PCV13 vaccination in a country with intermediate vaccination coverage. *Vaccine* **36**, 7744–7752 (2018).
9. Maraki, S., Mantadakis, E. & Samonis, G. Serotype distribution and antimicrobial resistance of adult Streptococcus pneumoniae clinical isolates over the period 2001-2008 in Crete, Greece. *Chemotherapy* **56**, 325–332 (2010).
10. Grau, I. *et al.* Smoking and alcohol abuse are the most preventable risk factors for invasive pneumonia and other pneumococcal infections. *International Journal of Infectious Diseases* **25**, 59–64 (2014).

11. Sanz, J. C. *et al.* Multidrug-resistant pneumococci (serotype 8) causing invasive disease in HIV+ patients. *Clinical Microbiology and Infection* **17**, 1094–1098 (2011).

12. Sanz, J. C., Rodríguez-Avial, I., Ríos, E., Ramos, B. & Cercenado, E. Recurrent pneumococcal invasive disease in the Region of Madrid during a five-year period. *Infection* **42**, 475–483 (2014).

13. Heckenberg, S. G. B., Brouwer, M. C., van der Ende, A., Hensen, E. F. & van de Beek, D. Hearing loss in adults surviving pneumococcal meningitis is associated with otitis and pneumococcal serotype. *Clinical Microbiology and Infection* **18**, 849–855 (2012).

14. de Miguel, S. *et al.* Nationwide Trends of Invasive Pneumococcal Disease in Spain From 2009 Through 2019 in Children and Adults During the Pneumococcal Conjugate Vaccine Era. *Clinical Infectious Diseases* **73**, e3778–e3787 (2021).

15. Gutiérrez Rodríguez angelesgutierrez, M. A. *et al. Surveillance and outbreak reports*. *Euro Surveill* vol. 19 www.eurosurveillance.org:pii=20922.Availableonline:http://www.eurosurveillance.org/ViewArticle.aspx?ArticleId=20922 (2008).

16. Iraurgui, P., Torres, M. J. & Aznar, J. Molecular epidemiology of fluoroquinolone resistance in invasive clinical isolates of Streptococcus pneumoniae in Seville. *Enferm Infecc Microbiol Clin* **30**, 180–183 (2012).

17. Ochoa-Gondar, O. & Vila-Corcoles, A. Prevalence of invasive pneumococcal disease among elderly people in Southern Catalonia, Spain, 2002-2009: An increase in serotypes not contained in the heptavalent conjugate vaccine. *Journal of Infection* **63**, 434–440 (2011).

18. Vestjens, S. M. T. *et al.* Twelve years of pneumococcal conjugate vaccination in the Netherlands: Impact on prevalence and clinical outcomes of invasive pneumococcal disease. *Vaccine* **37**, 6558–6565 (2019).

19. Horácio, A. N., Diamantino-Miranda, J., Aguiar, S. I., Ramirez, M. & Melo-Cristino, J. Serotype changes in adult invasive pneumococcal infections in Portugal did not reduce the high fraction of potentially vaccine preventable infections. *Vaccine* **30**, 218–224 (2012).

20. Wagenvoort, G. H. J. *et al.* Risk and outcomes of invasive pneumococcal disease in adults with underlying conditions in the post-PCV7 era, The Netherlands. *Vaccine* **34**, 334–340 (2016).

21. Horácio, A. N., Diamantino-Miranda, J., Aguiar, S. I., Ramirez, M. & Melo-Cristino, J. The Majority of Adult Pneumococcal Invasive Infections in Portugal Are Still Potentially Vaccine Preventable in Spite of Significant Declines of Serotypes 1 and 5. *PLoS One* **8**, (2013).

22. Sá-Leao, R. *et al.* Analysis of invasiveness of pneumococcal serotypes and clones circulating in portugal before widespread use of conjugate vaccines reveals heterogeneous behavior of clones expressing the same serotype. *J Clin Microbiol* **49**, 1369–1375 (2011).

23. Wagenvoort, G. H. J. *et al.* Invasive pneumococcal disease: Clinical outcomes and patient characteristics 2–6 years after introduction of 7-valent pneumococcal conjugate vaccine compared to the pre-vaccine period, the Netherlands. *Vaccine* **34**, 1077–1085 (2016).

24. Lambertsen, L., Brendstrup, M., Friis, H. & Christensen, J. J. Molecular characterization of invasive penicillin non-susceptible Streptococcus pneumoniae from Denmark, 2001 to 2005. *Scand J Infect Dis* **42**, 333–340 (2010).

25. Bewick, T. *et al.* Serotype prevalence in adults hospitalised with pneumococcal non-invasive community-acquired pneumonia. *Thorax* **67**, 540–545 (2012).

26. Horácio, A. N. *et al.* Population structure of Streptococcus pneumoniae causing invasive disease in adults in Portugal before PCV13 availability for adults: 2008-2011. *PLoS One* **11**, (2016).

27. Alari, A. *et al.* Impact of pneumococcal conjugate vaccines on pneumococcal meningitis cases in France between 2001 and 2014: A time series analysis. *BMC Med* **14**, (2016).

28. Ouldali, N. *et al.* Invasive pneumococcal disease prevalence in children and adults in France during the pneumococcal conjugate vaccine era: an interrupted time-series analysis of data from a 17-year national prospective surveillance study. *Lancet Infect Dis* **21**, 137–147 (2021).

29. Monali, R. *et al.* Impact of vaccination on invasive pneumococcal disease in Italy 2007-2017: Surveillance challenges and epidemiological changes. *Epidemiol Infect* (2020) doi:10.1017/S0950268820001077.

30. Nieddu, F. *et al.* PCV13 serotype decrease in Italian adolescents and adults in the post-PCV13 era: Herd protection from children or secular trend? *Vaccine* **35**, 1544–1550 (2017).

31. Imöhl, M. *et al.* Pneumococcal meningitis and vaccine effects in the era of conjugate vaccination: Results of 20 years of nationwide surveillance in Germany. *BMC Infect Dis* **15**, (2015).

32. Polkowska, A. *et al.* Pneumococcal meningitis before the introduction of 10-valent pneumococcal conjugate vaccine into the National Childhood Immunization Program in Poland. *Vaccine* **37**, 1365–1373 (2019).

33. van Hoek, A. J., Andrews, N., Waight, P. A., George, R. & Miller, E. Effect of serotype on focus and mortality of invasive pneumococcal disease: Coverage of different vaccines and insight into non-vaccine serotypes. *PLoS One* **7**, (2012).

34. Skoczyńska, A. *et al.* The current status of invasive pneumococcal disease in Poland. *Vaccine* **29**, 2199–2205 (2011).

35. Moore, C. E. *et al.* Reduction of invasive pneumococcal disease 3 years after the introduction of the 13-valent conjugate vaccine in the Oxfordshire region of England. *Journal of Infectious Diseases* **210**, 1001–1011 (2014).

36. Flasche, S. *et al.* Effect of pneumococcal conjugate vaccination on serotype-specific carriage and invasive disease in England: A cross-sectional study. *PLoS Med* **8**, 14 (2011).

37. Hughes, G. J., Wright, L. B., Chapman, K. E., Wilson, D. & Gorton, R. Serotype-specific differences in short- and longer-term mortality following invasive pneumococcal disease. *Epidemiol Infect* **144**, 2654–2669 (2016).

38. Chapman, K. E., Wilson, D. & Gorton, R. Serotype dynamics of invasive pneumococcal disease post-PCV7 and pre-PCV13 introduction in North East England. *Epidemiol Infect* **141**, 344–352 (2013).

39. Pichon, B. *et al.* Changes in molecular epidemiology of Streptococcus Pneumoniae causing meningitis following introduction of pneumococcal conjugate vaccination in England and Wales. *J Clin Microbiol* **51**, 820–827 (2013).

40. Miller, E., Andrews, N. J., Waight, P. A., Slack, M. P. E. & George, R. C. Herd immunity and serotype replacement 4 years after seven-valent pneumococcal conjugate vaccination in England and Wales: an observational cohort study. *Lancet Infect Dis* **11**, 760–768 (2011).

41. Lamb, K. E. *et al.* Trends in serotypes and sequence types among cases of invasive pneumococcal disease in Scotland, 1999-2010. *Vaccine* **32**, 4356–4363 (2014).

42. Vickers, I. *et al.* Serotype distribution of Streptococcus pneumoniae causing invasive disease in the Republic of Ireland. *Epidemiol Infect* **139**, 783–790 (2011).

43. Corcoran, M. *et al.* The epidemiology of invasive pneumococcal disease in older adults in the post-PCV era. Has there been a herd effect? *Epidemiol Infect* **145**, 2390–2399 (2017).

44. Bijlsma, M. W. *et al.* Community-acquired bacterial meningitis in adults in the Netherlands, 2006-14: A prospective cohort study. *Lancet Infect Dis* **16**, 339–347 (2016).

45. van Deursen, A. M. M. *et al.* Invasive pneumococcal disease and 7-valent pneumococcal conjugate vaccine, the Netherlands. *Emerg Infect Dis* **18**, 1729–1737 (2012).

46. van Werkhoven, C. H. *et al.* Pneumococcal conjugate vaccine herd effects on non-invasive pneumococcal pneumonia in elderly. *Vaccine* **34**, 3275–3282 (2016).

47. Hansen, C. B. *et al.* Molecular characterization and epidemiology of Streptococcus pneumoniae serotype 8 in Denmark. *BMC Infect Dis* **21**, (2021).

48. Slotved, H. C., Dalby, T. & Hoffmann, S. The effect of pneumococcal conjugate vaccines on the prevalence of invasive pneumococcal disease caused by ten non-vaccine serotypes in Denmark. *Vaccine* **34**, 769–774 (2016).

49. Ingels, H. *et al.* Impact of pneumococcal vaccination in Denmark during the first 3 years after PCV introduction in the childhood immunization programme. *Vaccine* **30**, 3944–3950 (2012).

50. Naucler, P. *et al.* Comparison of the Impact of Pneumococcal Conjugate Vaccine 10 or Pneumococcal Conjugate Vaccine 13 on Invasive Pneumococcal Disease in Equivalent Populations. *Clinical Infectious Diseases* **65**, 1780–1789 (2017).

51. Munson, S. *et al.* Clinical burden of pneumonia, meningitis and septicemia in Norway 2 years after 7-valent pneumococcal conjugate vaccine introduction. *Scand J Public Health* **43**, 657–666 (2015).

52. Vestrheim, D. F. *et al.* Indirect effect of conjugate pneumococcal vaccination in a 2 + 1 dose schedule. *Vaccine* **28**, 2214–2221 (2010).

53. Fenoll, A. *et al.* Variations in serotypes and susceptibility of adult non-invasive Streptococcus pneumoniae isolates between the periods before (May 2000-May 2001) and 10 years after (May 2010-May 2011) introduction of conjugate vaccines for child immunisation in Spain. *Int J Antimicrob Agents* **40**, 18–23 (2012).

54. Horácio, A. N., Lopes, J. P., Ramirez, M. & Melo-Cristino, J. Non-invasive pneumococcal pneumonia in Portugal - Serotype distribution and antimicrobial resistance. *PLoS One* **9**, (2014).

55. González-Díaz, A. *et al.* Two multi-fragment recombination events resulted in the β-lactam-resistant serotype 11A-ST6521 related to Spain9V-ST156 pneumococcal clone spreading in south-western Europe, 2008 to 2016. *Eurosurveillance* **25**, (2020).

56. Browall, S. *et al.* Clinical manifestations of invasive pneumococcal disease by vaccine and non-vaccine types. *European Respiratory Journal* **44**, 1646–1657 (2014).

57. Ríos Dueñas, E., Rodríguez-Avial, I. & Picazo, J. J. In vitro activity of ceftobiprole and seven other antimicrobial agents against invasive Streptococcus pneumoniae isolates in Spain. *European Journal of Clinical Microbiology and Infectious Diseases* **30**, 1621–1625 (2011).

58. Galanis, I. *et al.* Effects of PCV7 and PCV13 on invasive pneumococcal disease and carriage in Stockholm, Sweden. *European Respiratory Journal* **47**, 1208–1218 (2016).

59. Spijkerman, J. *et al.* Carriage of Streptococcus pneumoniae 3 Years after Start of Vaccination Program, the Netherlands . *Emerg Infect Dis* **17**, 584–591 (2011).

60. Dobay, O. *et al.* Genotypic and phenotypic characterisation of invasive Streptococcus pneumoniae isolates from Hungary, and coverage of the conjugate vaccines. *J Clin Pathol* **63**, 1116–1120 (2010).

61. Littorin, N., Ahl, J., Uddén, F., Resman, F. & Riesbeck, K. Reduction of Streptococcus pneumoniae in upper respiratory tract cultures and a decreased prevalence of related acute otitis media following introduction of childhood pneumococcal conjugate vaccines in a Swedish county. *BMC Infect Dis* **16**, (2016).

62. Quirk, S. J. *et al.* Vaccination of Icelandic Children with the 10-Valent Pneumococcal Vaccine Leads to a Significant Herd Effect among Adults in Iceland. *J Clin Microbiol* **57**, (2019).

63. Grall, N. *et al.* Epidemiology of Streptococcus pneumoniae in France before introduction of the PCV-13 vaccine. *European Journal of Clinical Microbiology and Infectious Diseases* **30**, 1511–1519 (2011).

64. Worsøe, L., Cayé-Thomasen, P., Brandt, C. T., Thomsen, J. & Østergaard, C. Factors associated with the occurrence of hearing loss after pneumococcal meningitis. *Clinical Infectious Diseases* **51**, 917–924 (2010).

65. Verhaegen, J. *et al.* Epidemiology and outcome of invasive pneumococcal disease among adults in Belgium, 2009–2011. *Eurosurveillance* **19**, (2014).

66. Marimon, J. M., Ercibengoa, M., García-Arenzana, J. M., Alonso, M. & Pérez-Trallero, E. Streptococcus pneumoniae ocular infections, prominent role of unencapsulated isolates in conjunctivitis. *Clinical Microbiology and Infection* **19**, (2013).

67. van der Linden, M., Perniciaro, S. & Imöhl, M. Increase of serotypes 15A and 23B in IPD in Germany in the PCV13 vaccination era. *BMC Infect Dis* **15**, (2015).

68. van der Linden, M., Falkenhorst, G., Perniciaro, S. & Imöhl, M. Effects of infant pneumococcal conjugate vaccination on serotype distribution in invasive pneumococcal disease among children and adults in germany. *PLoS One* **10**, (2015).

69. Ardanuy, C. *et al.* Epidemiology of invasive pneumococcal disease in older people in Spain (2007-2009): Implications for future vaccination strategies. *PLoS One* **7**, (2012).

70. Vila-Corcoles, A. *et al.* Pneumococcal pneumonia in adults 60 years or older: Prevalence, mortality and prevention. *Med Clin (Barc)* **146**, 199–202 (2016).

71. Richter, L. *et al.* Invasive pneumococcal diseases in children and adults before and after introduction of the 10-valent pneumococcal conjugate vaccine into the Austrian national immunization program. *PLoS One* **14**, (2019).

72. Foster, D. *et al.* Reduction in invasive pneumococcal disease following implementation of the conjugate vaccine in the Oxfordshire region, England. *J Med Microbiol* **60**, 91–97 (2011).

73. Rodenburg, G. D. *et al.* Effects of pneumococcal conjugate vaccine 2 years after its introduction, the Netherlands. *Emerg Infect Dis* **16**, 816–823 (2010).

74. Elberse, K. E. M. *et al.* Changes in the composition of the pneumococcal population and in IPD prevalence in The Netherlands after the implementation of the 7-valent pneumococcal conjugate vaccine. *Vaccine* **30**, 7644–7651 (2012).

75. Siira, L. *et al.* Antimicrobial resistance in relation to sero-and genotypes among invasive streptococcus pneumoniae in Finland, 2007-2011. *Microbial Drug Resistance* **20**, 124–130 (2014).

76. Calatayud, L. *et al.* Serotype and genotype replacement among macrolide-resistant invasive pneumococci in adults: Mechanisms of resistance and association with different transposons. *J Clin Microbiol* **48**, 1310–1316 (2010).

77. Siira, L., Vestrheim, D. F., Winje, B. A., Caugant, D. A. & Steens, A. Antimicrobial susceptibility and clonality of Streptococcus pneumoniae isolates recovered from invasive disease cases during a period with changes in pneumococcal childhood vaccination, Norway, 2004–2016. *Vaccine* **38**, 5454–5463 (2020).

Supplementary Table S4 - Summary of the findings from the PCV10/13 era (2010-2015, unless stated otherwise by the authors of each paper).

	18-64yo (IPD)	≥65yo (IPD	18-64yo (other)	≥65yo (other)	Severity and Lethality	Antimicrobial resistance
**8**	• 4.0% prevalence in Spain [1]*		• 2nd most prevalent serotype in adults hospitalized with CAP in the Netherlands [2]		• Caused IPD more frequently in Spanish smokers (OR 3.28) [3]	• 19.3% of serotype 8 isolates showed LVX non-susceptibility, and 22.9% showed ERY non-susceptibility (IPD, Spain) [4]
	• 7.7% prevalence in Spain – 3rd most prevalent serotype (13.4% prevalence in 2014/2015) [4]					
	• 4.1% prevalence in BPP in Spain [5]				• Significantly associated with lower CFR (IPD, Netherlands) [6]*	
	• 0.47 in 100.000 prevalence in Spain [7]		• 3.7% prevalence in NIPP in Portugal (2015) [8]			
	• Significantly decreased vs PCV7 era -35% (Spain) [7]				• Significantly lower odds of meningitis (OR 0.69, IPD, England and Wales) [9]*	
	• Main serotype causing recurrent IPD in Spain (12.1%) [10]*		• Among the serotypes with higher case:carrier ratio (prevalent in IPD but not in carriage) in England [11]*			
	• 14% prevalence in Spain [12]	• 8.9% prevalence in Spain [12]			• Significantly associated with increased odds of death (OR 2.9, CFR 33%) (IPD, England and Wales) [9]*	
	• 3.9% prevalence in Spain (2010) [13]*					
	• Most common serotype in young adults (<50yo) in Spain 10.4% [4]	• 2nd most common serotype in older adults in Spain 5.5% [4]		• Record of an unusual outbreak of LRTI due to pneumococcal serotype 8 with an exceptionally high attack rate (65%) in a residential home with elderly individuals vaccinated with the PPV23 [14]		
	• 5.0% prevalence in Catalonia, Spain [15]*				• Most common in sepsis not associated with other clinical conditions (IPD, Italy) 11.6% [16]	
	• 0.61 per 100.000 prevalence in Catalonia, Spain [17]*					
	• Significantly increased vs PCV7 era IRR 1.73 (Catalonia, Spain) [17]*					
	• 8.1% prevalence in Catalonia, Spain [18]*				• Higher prevalence in younger adults than older (15-64 vs ≥65yo, IPD, England) [19]	
		• 5.0% prevalence in individuals aged 60 and up, Madrid, Spain [20]			• More present in individuals with no comorbidities comparing to PCV13 serotypes (IPD, England) [19]	
	• Among the most common serotypes in Portugal (6.2%) [21]					
	• Significantly increased vs PCV7 era (8% vs 3.7%, Portugal) [21]				• Significantly lower CFR comparing to PCV13 serotypes OR 0.44 (IPD, England) [19]	
	• Significantly more frequent in the younger adults (18-49) 10.5% (Portugal) [21]	• vs 4.5% in ≥65yo [21]				
	• 9.1% prevalence in 18-49yo, 4.5% in 50-64yo in Portugal [22]	• 4.2% prevalence in Portugal [22]			• High CFP in meningitis in Poland 29% [23]*	
	• 3% prevalence in pneumococcal meningitis in France (2013-2014) [24]*					
	• 1.9% prevalence in France (2010) [13]*				• Significantly less common in high-risk group patients (IPD, Netherlands) [25]	
		• 2.3% prevalence in France [26]	• 1.6% of all-cause CAP in Germany [27]			
	• 7.9% prevalence in Italy (2014) [28]*				• Among most common serotypes (2nd) in pneumonia isolates 9.6% (IPD, Spain) –	
	• Among the most frequent serotypes in Italy 8.5% (+5.5% vs PCV7 era) [16]					

	18-64yo (IPD)	≥65yo (IPD	18-64yo (other)	≥65yo (other)	Severity and Lethality	Antimicrobial resistance
	• 2.5% prevalence in Italy (2010) [13]*		• 8.1% prevalence in CAP in England - 2nd most prevalent serotype [29]		no difference in prevalence between uncomplicated and complicated pneumonia [4]	
	• 28.6% prevalence in Malta (2010) [13]*		• 18.3% prevalence in CAP in the UK [30]			
	• 3.0% prevalence in Belgium (2010) [13]*					
	• 3.3% pneumococcal meningitis isolates in Germany [31]		• Identified in 7.7% of the young adults who were carriers in the UK [32]		• Significantly more frequent in immunocompetent patients 9.3% vs 5.6% (IPD, Spain) [4]	
	• Significantly increasing vs PCV7 era in IPD in Germany (4.0% vs 3.0%) [33].					
	• *21.1% of bacteremic pneumococcal CAP in Germany [27]*					
	• 2.4% prevalence in Poland (2010) [13]*					
	• 5.4% prevalence in Poland (2015) [23]*					
	• 4.8% (15-24yo), 3.9% (25-54yo), 4.6% (55-64yo) prevalence in Poland [34]	• 1.0% prevalence in Poland [34]				
	• 1.7% prevalence in Czech Republic (2010) [13]*			• Identified in 16.7% of the older adults who were carriers in Italy [35]		
	• 1.7% prevalence in Austria (2010) [13]*					
	• 0.9% prevalence in Hungary (2010) [13]*					
	• 1.3% prevalence in Slovenia (2010) [13]*					
	• 3.2% prevalence in Serbia (2009-2016) [36]	• 7.4% prevalence in Serbia (2009-2016) [36]				
	• 20.2% prevalence in England [37]*					
	• 30.3% prevalence (15-44yo), 25.7% prevalence (45-64yo) in England [19]	• 18.6% prevalence (65-79yo), 10.0% prevalence (>80yo) in England [19]				
	• 7.5% prevalence in North East England [38]*					
	• Significantly increased vs PCV7 era IRR 1.33 in England and Wales [39][$]	• Significantly increased vs PCV7 era IRR 1.43 in England and Wales [39]				
	• Significantly increasing vs PCV7 era in Scotland (OR 1.16) [40]*					
		• 2.0 per 100.000 prevalence in Ireland (2012-2013) [41]				
	• 9.3% prevalence in Ireland (2010) [13]*					
	• 9.1% prevalence in the UK (2010) [13]*					
	• Prevalence in the Netherlands 1.5 per 100.000 [6]*					
	• 8% prevalence in community acquired bacterial (pneumococcal) meningitis in the Netherlands [42]					
		• 6.7 per 100.000 prevalence in the Netherlands [43]				

	18-64yo (IPD)	≥65yo (IPD	18-64yo (other)	≥65yo (other)	Severity and Lethality	Antimicrobial resistance
		• 12% prevalence in the Netherlands [44]				
	• 6.7% prevalence in the Netherlands (2010) [13]*					
	• 8.3% prevalence in bacteremic pneumonia in Denmark (vs 1.8% prevalence in non-bacteremic pneumonia) [45]					
	• Mean prevalence in Denmark 0.91 [46][$]	• Mean prevalence in Denmark 4.87 [46][$]				
	• 5.9% prevalence in Denmark (2010) [13]*					
	• 0.42 per 100.000 prevalence in Sweden (2014) [47]*					
	• 2.6% prevalence in Norway (2010) [13]*					
	• 0.6% prevalence in Finland (2010) [13]*					
**10A**	• 1.8% prevalence in Spain [1]*		• 2.3% prevalence in NIPP in Portugal (2015) [8]		• Significantly higher odds of meningitis (OR 2.06, IPD, England and Wales) [9]*	• 31.8% of serotype 10A isolates showed ERY non-susceptibility (IPD, Spain) [4]
	• 2.0% prevalence in Spain (2.9% prevalence in 2014/2015) [4]					
	• 0.11 in 100.000 prevalence in Spain [7]		• 3.0% carriage across several countries in Europe (Austria, Belgium, Croatia, France, Hungary, Netherlands, Spain, Sweden, UK) [48]*		• High CFP in meningitis in Poland 22% [23]*	
	• 1.8% prevalence in Spain (2010) [13]*					
	• 3.1% prevalence in Catalonia, Spain [18]*					
	• 0.24 per 100.000 prevalence in Catalonia, Spain [17]*				• Significantly more common in high-risk group patients 39% vs 16% (average) – significantly associated with IPD in high-risk patients (Netherlands) [25]	
	• 2.1% prevalence in Catalonia, Spain [15]*					
	• 6% prevalence in pneumococcal meningitis in France (2013-2014) - among most frequent non-vaccine serotypes [24]*		• Lower carriage in Austria, Croatia and Sweden (0%) and higher in UK (13.9%) [48]			
	• 2.5% prevalence in France (2010) [13]*				• Significantly more frequent in immunocompromised patients 3.6% vs 0.8% (IPD, Spain) [4]	
		• 2.5% prevalence in France [26]				
	• 3.1% prevalence in Italy (2014) [28]*					
	• 1.8% prevalence in Italy (2010) [13]*				• Pneumonia diagnosed rarely among cases caused by serotype 10A 5.0% (IPD, Poland) [34]	
	• 0.8% prevalence in Belgium (2010) [13]*					
	• 4.0% pneumococcal meningitis isolates in Germany [31]					
	• Significantly increasing vs PCV7 era in IPD in Germany (3.0% vs 2.3%) [33]					
	• Prevalence was significantly higher than would have been expected without vaccination (2014-2015, Poland) [23]*					
	• 7.5% prevalence in Poland (2015) [23]*					
	• 1.5% prevalence in Poland (2010) [13]*					
	• 2.9% (25-54yo) prevalence in Poland [49]	• 1.9% prevalence in Poland [49]				
	• 2.7% prevalence in Czech Republic (2010) [13]*					
	• 1.4% prevalence in Austria (2010) [13]*					

	18-64yo (IPD)	≥65yo (IPD	18-64yo (other)	≥65yo (other)	Severity and Lethality	Antimicrobial resistance
	• 0.9% prevalence in Hungary (2010) [13]*					
	• 2.2% prevalence in Slovenia (2010) [13]*					
	• 3.3% prevalence in England [37]*					
	• 1.2% prevalence in North East England [38]*					
		• Significantly increased vs PCV7 era IRR 2.58 in England and Wales [39]				
	• 2.0% prevalence in the UK (2010) [13]*					
	• 1.2% prevalence in Ireland (2010) [13]*					
	• 4.4% prevalence in the Netherlands (2010) [13]*					
	• 2.1% prevalence in Denmark (2010) [13]*					
	• 0.37 per 100.000 prevalence in Sweden (2014) [47]*					
	• Significantly increased vs PCV7 era RR 2.84 (Sweden) [50]*					
	• 1.9% prevalence in Norway (2010) [13]*					
	• 0.4% prevalence in Finland (2010) [13]*					
**11A**	• 2.4% prevalence in Spain [1]*		• Among the most common serotypes in NIPP in Portugal (6.7%) [51]		• Caused IPD more frequently in Spanish alcohol abusers (OR 3.28) [3]	• Increases in non-susceptibility to PEN and ERY [1]*
	• 4.2% prevalence in Spain – 7th most prevalent serotype (5.3% prevalence in 2014/2015) [4]					
	• 0.34 in 100.000 prevalence in Spain [7]				• Significantly associated with higher CFR (IPD, Netherlands) [6]*	• Among the most frequent serotypes showing ERY resistance 16.3% (emerging in the PCV10/13 era, IPD, Spain) [7]
	• 2.1% prevalence in Spain (2008) [52]		• Identified in 7.7% of the young adults who were carriers in the UK [32]			
	• 1.8% prevalence in Spain (2010) [13]*				• IPD patients with serotype 11A were significantly older than those infected with other vaccinal serotypes (Sweden) [50]*	
	• 3.3% prevalence in Spain (2012) [52]					
		• 3.1% of the invasive isolates in Majorca, Spain (14.3% total isolates) [53]			• Serotype isolated more frequently among adults than children (IPD, Spain) [52]	
						• 69.9% of serotype 11A isolates showed PEN non-susceptibility, 50% showed CTX non-susceptibility, and 28.3% showed ERY non-susceptibility (IPD, Spain) [4]
		• 3.8% prevalence in individuals aged 60 and up, Madrid, Spain [20]	• Significantly associated with NIPP vs IPD (Portugal) [51]		• Among most common serotypes (2nd) in meningitis isolates 7.4% (IPD, Spain) [4]	
	• 1.9% prevalence in Catalonia, Spain [15]*					
	• 2.6% prevalence in Catalonia, Spain [18]*		• 9.2% prevalence in NIPP in Portugal (2015) – 2nd most prevalent serotype [8]		• Among most common serotypes (3rd) in non-focal bacteremia isolates 6.9% (IPD, Spain) [4]	
	• 0.23 per 100.000 prevalence in Catalonia, Spain [17]*					
	• Significantly increased vs PCV7 era IRR 2.35* 204%$^{\$}$ (Catalonia, Spain) [17]					
	• 2.8% prevalence in 18-49yo, 3.4% in 50-64yo in Portugal [22]	• 2.5% prevalence in Portugal [22]	• The proportion of serotype 11A associated with non-invasive pneumococcal disease increased		• Most common serotype in peritonitis isolates 14.7% (IPD, Spain) [4]	• 24% of isolates non-susceptible to ERY and 4% MDR (IPD, Poland) [34]
	• 3% prevalence in pneumococcal meningitis in France (2013-2014) [24]*					

	18-64yo (IPD)	≥65yo (IPD	18-64yo (other)	≥65yo (other)	Severity and Lethality	Antimicrobial resistance
	• 1.6% prevalence in France (2010) [13]*		in Spanish adults during the period 2000-2012 (from 3.3% to 9.3%) [52]		• Significantly more frequent in immunocompromised patients 5.8% vs 3.1% (IPD, Spain) [4]	• The proportion of highly penicillin-resistant (MIC > 2 ug/mL) serotype 11A isolates corresponded to 23.8% in 2012 (increased vs previous years, IPD, Spain) [52]
	• 1.9% prevalence in Italy (2014) [28]*					
	• 0.7% prevalence in Italy (2010) [13]*		• 3.5% prevalence in CAP in the UK [30]		• Highest CFR 52.2% (IPD, Poland) [34]	
					• Serotype isolated more frequently among adults than children (IPD, Spain) [52]	
	• 0.6% prevalence in Belgium (2010) [13]*			• 14.3% of the isolates in Majorca, Spain but only 3.1% of the invasive isolates – more associated with non-invasive disease [53]	• Combined data from several European countries found that serotype 11A is significantly associated with death RR 1.97 [54]*	
	• 4.0% pneumococcal meningitis isolates in Germany [55].					
	• 1.1% prevalence in Poland (2015) [23]*					
	• 2.1% (25-54yo), 3.0% (55-64yo) prevalence in Poland [34]	• 2.2% prevalence in Poland [34]				
	• 2.9% prevalence in Poland (2010) [13]*					
	• 0.7% prevalence in Austria (2010) [13]*					
	• 0.18 per 100.000 prevalence in Austria (rank 9) [56]					
	• 1.7% prevalence in Czech Republic (2010) [13]*					
	• 3.7% prevalence in Hungary (2010) [13]*					
	• 1.3% prevalence in Slovenia (2010) [13]*		• Among the most prevalent serotypes in carriage in individuals over 60 years in Portugal 6.4% [57]			
	• 3.3% prevalence in England [37]*					
	• 1.7% prevalence in North East England [38]*					
		• 0.55 per 100.000 prevalence in Ireland (2012-2013) [41]				
			• Most common in carriage accross several countries in Europe (Austria, Belgium, Croatia, France, Hungary, Netherlands, Spain, Sweden, UK) 6.4% [48]*			
	• 0.6% prevalence in the UK (2010) [13]*					
	• 2.4% prevalence in Ireland (2010) [13]*					
		• 1.7% prevalence in the Netherlands [44][#]				
	• Mean prevalence in Denmark 0.13 [46][$]	• Mean prevalence in Denmark 1.28 [46][$]				
	• 2.6% prevalence in Denmark (2010) [13]*		• Lower carriage in Croatia (2.2%) and Spain (2.4%), higher in France (10.6%) and Sweden (10.7%) [48]*			
	• 0.34 per 100.000 prevalence in Sweden (2014) [47]*					
	• Significantly increase vs the PCV7 era RR 1.74 (Sweden) [50]*					
	• 10.0% prevalence in acute otitis media in Sweden – most prevalent serotype [58]*		• 7.7% prevalence in non-bacteremic pneumonia in			
	• Significantly increased vs PCV7 era (acute otitis media, Sweden) [58]*					

	18-64yo (IPD)	≥65yo (IPD	18-64yo (other)	≥65yo (other)	Severity and Lethality	Antimicrobial resistance
	• 3.2% prevalence in Norway (2010) [13]*		Denmark (vs 1.0% prevalence in bacteremic pneumonia) [45]			
	• 1.6% prevalence in Finland (2010) [13]*					
	• 4.0 per 100.000 prevalence in Iceland [59]	• 17.1 per 100.000 prevalence in Iceland [59]	• 0.8% of all-cause CAP in Germany [27]			
				• Identified in 8.3% of the older adults who were carriers in Italy [35]		
**12F**	• 3.5% prevalence in Spain [1]*		• 6th most prevalent serotype in adults hospitalized with CAP in the Netherlands [2]		• Among the most frequent serotypes in septic arthritis (UK) [60]	
	• 3.2% prevalence in Spain (5.3% prevalence in 2014/2015) [4]					
	• 0.45 in 100.000 prevalence in Spain [7]				• Significantly associated with lower CFR (IPD, Netherlands) [6]*	
	• 2.7% prevalence in Spain (2010) [13]*		• 2.5% prevalence in CAP in England - 2nd most prevalent serotype [29]			
	• 3.4% prevalence in Spain [12]	• 3.5% prevalence in Spain [12]				
		• 3.1% of the invasive isolates in Majorca, Spain (2.9% total isolates) [53]	• 5.6% prevalence in CAP in the UK [30]		• Significantly less common in high-risk group patients (IPD, Netherlands) [25]	
	• 6.4% prevalence in Catalonia, Spain (9.2% in 2012) [15]*				• CFR 6% (IPD, Belgium) [61]#	
	• 8.1% prevalence in Catalonia, Spain [18]*				• Higher prevalence in younger adults than older (15-64 vs ≥65yo, IPD, England) [19]	
	• 0.79 per 100.000 prevalence in Catalonia, Spain [17]*				• More present in individuals with no comorbidities comparing to PCV13 serotypes (IPD, England) [19]	
	• Significantly increased vs PCV7 era IRR 1.45 (Catalonia, Spain) [17]					
	• 9.3% prevalence in Catalonia, Spain [15]	•				
	• 7% prevalence in pneumococcal meningitis in France (2013-2014) – most frequent non-vaccine serotype [24]*				• Significantly lower CFR comparing to PCV13 serotypes OR 0.54 (IPD, England) [19]	
	• 7.1% prevalence in France (2010) [13]*					
		• 6.5% prevalence in France [26]				
	• 7.9% prevalence in Italy (2014) [28]*					
	• 1.1% prevalence in Italy (2010) [13]*					
	• 8% prevalence in Belgium, 5th most prevalent serotype [61]					
	• 2.7% prevalence in Belgium (2010) [13]*					
	• 5.3% of bacteremic pneumococcal CAP in Germany [27]					

	18-64yo (IPD)	≥65yo (IPD	18-64yo (other)	≥65yo (other)	Severity and Lethality	Antimicrobial resistance
	• 5.9% pneumococcal meningitis isolates in Germany [31].					
	• Among most strongly increasing non-PCV13 serotypes in the PCV10/13 era in IPD in Germany [62].					
	• Significantly increasing vs PCV7 era in IPD in Germany (5.8% vs 1.9%) [33].					
	• Prevalence was significantly higher than would have been expected without vaccination (2014-2015, Poland) [63]*					
	• 2.2% prevalence in Poland (2015) [23]*					
	• 2.9% prevalence in Poland (2010) [13]*					
	• 3.9% (25-54yo), 2.1% (55-64yo) prevalence in Poland [34]	• 1.9% prevalence in Poland [34]				
	• 1.0% prevalence in Czech Republic (2010) [13]*					
	• 0.9% prevalence in Hungary (2010) [13]*					
	• 1.0% prevalence in Austria (2010) [13]*					
	• 8.0% prevalence in England [37]*					
	• 2.9% prevalence in North East England [38]*					
	• 19.4% prevalence (15-44yo), 15.4% prevalence (45-64yo) in England [19]	• 9.4% prevalence (65-79yo), 6.4% prevalence (>80yo) in England [19]				
		• Significantly increased vs PCV7 era IRR 2.12 in England and Wales [39]				
		• 0.36 per 100.000 prevalence in Ireland (2012-2013) [41]				
	• 2.0% prevalence in the UK (2010) [13]*					
	• 2.8% prevalence in Ireland (2010) [13]*					
		• 2.6% prevalence in the Netherlands [44]#				
	• Mean prevalence in Denmark 0.51 [46]$	• Mean prevalence in Denmark 2.60 [46]$				
	• 4.2% prevalence in bacteremic pneumonia in Denmark (vs 1.1% prevalence in non-bacteremic pneumonia) [45]					
	• 4.1% prevalence in Denmark (2010) [13]*					
	• 0.13 per 100.000 prevalence in Sweden (2014) [47]*					
	• 0.3% prevalence in Norway (2010) [13]*					
	• 0.3% prevalence in Finland (2010) [13]*					
**15B**	• 0.07 in 100.000 prevalence in Spain [7]		• 2.3% prevalence in NIPP in Portugal (2015) [8]		• Significantly associated with higher CFR (IPD, Netherlands) [6]*	• 25% of serotype 15B isolates showed ERY non-susceptibility (IPD, Spain) [4]
	• 1.0% prevalence in Spain (2010) [13]*					
	• 1.2% prevalence in Catalonia, Spain [15]*					

	18-64yo (IPD)	≥65yo (IPD	18-64yo (other)	≥65yo (other)	Severity and Lethality	Antimicrobial resistance
	• 0.13 per 100.000 prevalence in Catalonia, Spain [17]*			• 2.9% of the isolates in Majorca, Spain (not detected in invasive isolates) [53]	• Significantly more common in high-risk group patients 52% vs 16% (average) – significantly associated with IPD in high-risk patients (Netherlands) [25]	• 20% of isolates non-susceptible to ERY (IPD, Poland) [34]
	• 1% prevalence in pneumococcal meningitis in France (2013-2014) [24]*					
	• 1.5% prevalence in France (2010) [13]*					
		• 1.7% prevalence in France [26]&			• Meningitis presentation more prevalent in patients infected with pneumococci of serotype 15B/C 63.3% vs 31.3% (IPD, Poland) [34]	
	• 1.5% prevalence in Italy (2014) [28]*					
	• 0.4% prevalence in Italy (2010) [13]*					
	• 0.4% prevalence in Belgium (2010) [13]*		• 3.7% carriage accross several countries in Europe (Austria, Belgium, Croatia, France, Hungary, Netherlands, Spain, Sweden, UK) [48]*&			
	• 3.0% pneumococcal meningitis isolates in Germany [31]				• Pneumonia diagnosed rarely among cases caused by serotype 15B/C 3.3% (IPD, Poland) [34]	
	• 2.9% (25-54yo), 2.1% (55-64yo) prevalence in Poland [34]	• 1.1% prevalence in Poland [34]				
	• 2.2% prevalence in Poland (2015) [23]*&					
	• 1.0% prevalence in Poland (2010) [13]*			• Identified in 8.3% of the older adults who were carriers in Italy [35]		
	• 0.7% prevalence in Czech Republic (2010) [13]*					
	• 1.7% prevalence in Austria (2010) [13]*					
	• 1.9% prevalence in Hungary (2010) [13]*					
	• 1.8% prevalence in Slovenia (2010) [13]*					
	• 2.7% prevalence in England [37]*					
	• 0.8% prevalence in the UK (2010) [13]*					
	• 1.2% prevalence in Ireland (2010) [13]*					
	• 2.2% prevalence in the Netherlands (2010) [13]*					
	• 0.9% prevalence in Denmark (2010) [13]*					
	• 0.16 per 100.000 prevalence in Sweden (2014) [47]*					
	• 9.3% prevalence in acute otitis media in Sweden – among the most prevalent serotypes [58]*					
	• Significantly increased vs PCV7 era (acute otitis media, Sweden) [58]*					
	• 1.3% prevalence in Norway (2010) [13]*					
	• 0.3% prevalence in Finland (2010) [13]*					
	• 2.0 per 100.000 prevalence in Iceland [59]&	• 27.4 per 100.000 prevalence in Iceland [59]&				
**22F**	• 4.4% prevalence in Spain [1]*		• Among the most common serotypes in NIPP in Portugal (4.1%) [51]		• Among the most frequent serotypes in septic arthritis (UK) [60]	
	• 4.2% prevalence in Spain – 8th most prevalent serotype [4]					
	• 6.5% prevalence in BPP in Spain [5]					

	18-64yo (IPD)	≥65yo (IPD	18-64yo (other)	≥65yo (other)	Severity and Lethality	Antimicrobial resistance
	• 0.39 in 100.000 prevalence in Spain [7]		• Among the most prevalent serotypes in carriage in individuals over 60 years in Portugal 9.0% [57]		• Significantly higher odds of meningitis (OR 1.39, IPD, England and Wales) [9]*	
	• 3.7% prevalence in Spain (2010) [13]*				• High CFP in meningitis in Poland 22% [23]*	
	• 4.3% prevalence in Spain [12]	• 6.1% prevalence in Spain [12]				
		• 4th most common serotype in older adults in Spain 5.4% [4]	• 4.8% prevalence in NIPP in Portugal (2015) [8]		• Percentage of septic shock was significantly higher in patients with serotype 22F in BPP [5]	
		• 9.4% of the invasive isolates in Majorca, Spain (8.6% total isolates) [53]			• CFR 12% (IPD, Belgium) [61]	
			• 3.4% carriage across several countries in Europe (Austria, Belgium, Croatia, France, Hungary, Netherlands, Spain, Sweden, UK) [48]*		• 2nd highest CFR 37.5% (IPD, Poland) [34]	
	• 4.3% prevalence in Catalonia, Spain [15]*					
	• 3.6% prevalence in Catalonia, Spain [18]*				• Higher prevalence in older adults than younger age groups, particularly ≥80 yo (IPD, England) [64]	
	• 0.53 per 100.000 prevalence in Catalonia, Spain [17]*					
	• Significantly increased vs PCV7 era IRR 5.34* 396%[$] (Catalonia, Spain) [17]					
		• 4.9% prevalence in individuals aged 60 and up, Madrid, Spain [20]	• 3.6% prevalence in non-bacteremic pneumonia in Denmark [45]		• Comorbidity prevalence of 68.7% - significantly higher than other serotypes altogether (IPD, England) [64]	
	• 3.2% prevalence in 18-49yo, 3.4% in 50-64yo in Portugal [22]	• 4.6% prevalence in Portugal [22]	• 1.0% of all-cause CAP in Germany [27]		• CFR 15.4% - significantly lower than other serotypes altogether (IPD, England) [64]	
	• 5% prevalence in pneumococcal meningitis in France (2013-2014) [24]*		• 3.3% prevalence in CAP in the UK [30]		• Death in 30d in patients with the 22F serotype associated with increasing age and clinical presentation with meningitis or septicaemia vs invasive pneumonia [64]	
	• 3.2% prevalence in France (2010) [13]*					
		• 6.8% prevalence in France [26]				
	• 4.0% prevalence in Italy (2014) [28]*					
	• 3.3% prevalence in Italy (2010) [13]*					
	• 1.7% prevalence in Belgium (2010) [13]*					
	• 5% prevalence in Belgium, 6th most prevalent serotype [61]					
	• 5.9% pneumococcal meningitis isolates in Germany [31]					
	• Among most strongly increasing non-PCV13 serotypes in the PCV10/13 era in IPD in Germany [62].					
	• Significantly increasing vs PCV7 era in IPD in Germany (6.9% vs 4.7%) [33].					

	18-64yo (IPD)	≥65yo (IPD	18-64yo (other)	≥65yo (other)	Severity and Lethality	Antimicrobial resistance
	• Prevalence was significantly higher than would have been expected without vaccination (2014-2015, IPD, Poland) [63]*					
	• 5.4% prevalence in Poland (2015) [23]*					
	• 2.1% (25-54yo), 1.7% (55-64yo) prevalence in Poland [34]	• 3.0% prevalence in Poland [34]				
	• 1.5% prevalence in Poland (2010) [13]*					
	• 1.7% prevalence in Czech Republic (2010) [13]*					
	• 3.1% prevalence in Austria (2010) [13]*					
	• 0.22 per 100.000 prevalence in Austria (rank 8) [56]					
	• 0.9% prevalence in Hungary (2010) [13]*					
	• 1.3% prevalence in Slovenia (2010) [13]*					
	• 14.3% prevalence in England [37]*					
	• 7.0% prevalence in England [64]					
	• Increased in prevalence after PCV7 era, plateaued in PCV10/13 era (England) [64]					
	• 16.5% prevalence in England [19]*					
	• 6.9% prevalence in North East England [38]*					
	• Significantly decreased vs PCV7 era IRR 0.65 in England and Wales [39]$					
	• Significantly increasing vs PCV7 era in Scotland (OR 1.51) [40]*					
		• 3.09 per 100.000 prevalence in Ireland (2012-2013) [41]				
	• 7.7% prevalence in Ireland (2010) [13]*					
	• 7.2% prevalence in the UK (2010) [13]*					
	• 7% prevalence in community acquired bacterial (pneumococcal) meningitis in the Netherlands [42]					
		• 4.1 per 100.000 prevalence in the Netherlands [43]				
		• 9.4% prevalence in the Netherlands [44]#				
	• 3.3% prevalence in bacteremic pneumonia in Denmark [45]					
	• 5.8% prevalence in Denmark (2010) [13]*					
	• Mean prevalence in Denmark 0.44 [46]$	• Mean prevalence in Denmark 3.80 [46]$				
	• 1.4 per 100.000 prevalence in Sweden (2014) [47]*					
	• Significantly increased vs PCV7 era RR 2.78 (Sweden) [50]*					
	• 12.4% prevalence in Norway (2010) [13]*					

	18-64yo (IPD)	≥65yo (IPD	18-64yo (other)	≥65yo (other)	Severity and Lethality	Antimicrobial resistance
	• 5.8% prevalence in Finland (2010) [13]*					
	• 8.8% of the IPD isolates in Finland (2011) – increased vs PCV7 era [65]*					
	• 4.7 per 100.000 prevalence in Iceland [59]	• 6.9 per 100.000 prevalence in Iceland [59]				
**33F**	• 0.11 in 100.000 prevalence in Spain [7]		• 0.7% prevalence in NIPP in Portugal (2015) [8]		• Significantly more common in high-risk group patients 30% vs 16% (average) – significantly associated with IPD in high-risk patients (Netherlands) [25]	• Emerged in Norway as a frequent serotype displaying resistance to several antimicrobials in IPD (ERY, SXT, CLI) [66]*
	• 1.3% prevalence in Spain (2010) [13]*					
	• 1.0% prevalence in Catalonia, Spain [15]*		• 2.1% prevalence in non-bacteremic pneumonia in Denmark [45]			
	• 1.6% prevalence in Catalonia, Spain [18]*					
	• 0.14 per 100.000 prevalence in Catalonia, Spain [17]*				• Significantly associated with IPD in high-risk patients (Netherlands) [25]	• Still among the most frequent serotypes showing ERY resistance 11.6% (IPD, Spain) [7]
	• Significantly increased vs PCV7 era IRR 1.74 (Catalonia, Spain) [17]					
		• 3.1% of the invasive isolates in Majorca, Spain (4.3% total isolates) [53]			• Highest prevalence in adults aged 65-79 (IPD, England) [64]	
	• 3% prevalence in pneumococcal meningitis in France (2013-2014) [24]*					
	• 1.1% prevalence in France (2010) [13]*				• Comorbidity prevalence of 67.2% - significantly higher than other serotypes altogether (IPD, England) [64]	
	• 2.1% prevalence in Italy (2014) [28]*					
	• 0.4% prevalence in Italy (2010) [13]*					
	• 1.1% prevalence in Belgium (2010) [13]*				• CFR 16.5% - significantly lower than other serotypes altogether (IPD, England) [64]	
	• 5.3% of bacteremic pneumococcal CAP in Germany [27]					
	• 1.9% pneumococcal meningitis isolates in Germany [31]				• Death in 30d in patients with the 33F serotype associated with increasing age, comorbidity status and clinical presentation with septicaemia vs invasive pneumonia [64]	
	• 0.5% prevalence in Poland (2010) [13]*					
	• 1.4% prevalence in Czech Republic (2010) [13]*					
	• 0.3% prevalence in Austria (2010) [13]*					
	• 1.0% prevalence in Hungary (2010) [13]*					
	• 7.6% prevalence in England [37]*					
	• 8.6% prevalence in England [19]*					
	• 3.5% prevalence in England [64]					
	• Increased in prevalence after PCV7 era, plateaued in PCV10/13 era (England) [64]					
	• Significantly lower CFR comparing to PCV13 serotypes OR 0.44 (IPD, England) [19]					
	• 2.6% prevalence in North East England [38]*					
		• 0.73 per 100.000 prevalence in Ireland (2012-2013) [41]				
	• 2.8% prevalence in Ireland (2010) [13]*					

	18-64yo (IPD)	≥65yo (IPD	18-64yo (other)	≥65yo (other)	Severity and Lethality	Antimicrobial resistance
	• 3.4% prevalence in the UK (2010) [13]*					
		• 2.6% prevalence in the Netherlands [44]#				
	• 2.6% prevalence in bacteremic pneumonia in Denmark [45]					
	• 2.3% prevalence in Denmark (2010) [13]*					
	• Mean prevalence in Denmark 0.15 [46]$	• Mean prevalence in Denmark 1.67 [46]$				
	• 0.65 per 100.000 prevalence in Sweden (2014) [47]*					
	• 3.3% prevalence in Norway (2010) [13]*					
	• 2.0 per 100.000 prevalence in Iceland [59]					

* total population, $ 5-64 years, & 15B/C

BPP bacteremic pneumococcal community-acquired pneumonia, CFP case fatality proportion, CFR case fatality ratio, CLI clindamycin, CTX cefotaxime, ERY erythromycin, IRR prevalence rate ratio, LVX levofloxacin, (N)IPP (non-)invasive pneumococcal pneumonia, OR odds ratio, PEN penicillin, SXT trimethoprim/sulfamethoxazole

References

1. Fenoll, A., Granizo, J. J., Giménez, M. J., Yuste, J. & Aguilar, L. Secular trends (1990-2013) in serotypes and associated non-susceptibility of S. pneumoniae isolates causing invasive disease in the pre-/post-era of pneumococcal conjugate vaccines in Spanish regions without universal paediatric pneumococcal vaccination. *Vaccine* **33**, 5691–5699 (2015).

2. Vestjens, S. M. T. *et al.* Changes in pathogens and pneumococcal serotypes causing community-acquired pneumonia in The Netherlands. *Vaccine* **35**, 4112–4118 (2017).

3. Grau, I. *et al.* Smoking and alcohol abuse are the most preventable risk factors for invasive pneumonia and other pneumococcal infections. *International Journal of Infectious Diseases* **25**, 59–64 (2014).

4. Fenoll, A. *et al.* Serotypes and genotypes of S. pneumoniae isolates from adult invasive disease in Spain: A 5-year prospective surveillance after pediatric PCV13 licensure. The ODIN study. *Vaccine* **36**, 7993–8000 (2018).

5. España, P. P. *et al.* Evolution of serotypes in bacteremic pneumococcal adult pneumonia in the period 2001–2014, after introduction of the pneumococcal conjugate vaccine in bizkaia (spain). *Vaccine* **37**, 3840–3848 (2019).

6. Vestjens, S. M. T. *et al.* Twelve years of pneumococcal conjugate vaccination in the Netherlands: Impact on prevalence and clinical outcomes of invasive pneumococcal disease. *Vaccine* **37**, 6558–6565 (2019).

7. Càmara, J. *et al.* Decrease of invasive pneumococcal disease (IPD) in adults after introduction of pneumococcal 13-valent conjugate vaccine in Spain. *PLoS One* **12**, (2017).

8. Horácio, A. N., Silva-Costa, C., Lopes, E., Ramirez, M. & Melo-Cristino, J. Conjugate vaccine serotypes persist as major causes of non-invasive pneumococcal pneumonia in Portugal despite declines in serotypes 3 and 19A (2012-2015). *PLoS One* **13**, (2018).

9. Oligbu, G. *et al.* Effect of pneumococcal conjugate vaccines on pneumococcal meningitis, England and Wales, July 1, 2000-June 30, 2016. *Emerg Infect Dis* **25**, 1708–1718 (2019).

10. Sanz, J. C., Rodríguez-Avial, I., Ríos, E., Ramos, B. & Cercenado, E. Recurrent pneumococcal invasive disease in the Region of Madrid during a five-year period. *Infection* **42**, 475–483 (2014).

11. van Hoek, A. J. *et al.* Pneumococcal carriage in children and adults two years after introduction of the thirteen valent pneumococcal conjugate vaccine in England. *Vaccine* **32**, 4349–4355 (2014).

12. de Miguel, S. *et al.* Nationwide Trends of Invasive Pneumococcal Disease in Spain From 2009 Through 2019 in Children and Adults During the Pneumococcal Conjugate Vaccine Era. *Clinical Infectious Diseases* **73**, e3778–e3787 (2021).

13. Torné, A. N. *et al.* European enhanced surveillance of invasive pneumococcal disease in 2010: Data from 26 European countries in the post-heptavalent conjugate vaccine era. *Vaccine* **32**, 3644–3650 (2014).

14. Thomas, H. L. *et al.* An explosive outbreak of Streptococcus pneumoniae serotype-8 infection in a highly vaccinated residential care home, England, summer 2012. *Epidemiol Infect* **143**, 1957–1963 (2015).

15. del Amo, E. *et al.* Serotypes and clonal diversity of streptococcus pneumoniae causing invasive disease in the Era of PCV13 in Catalonia, Spain. *PLoS One* **11**, (2016).

16. Nieddu, F. *et al.* PCV13 serotype decrease in Italian adolescents and adults in the post-PCV13 era: Herd protection from children or secular trend? *Vaccine* **35**, 1544–1550 (2017).

17. Ciruela, P. *et al.* The changing epidemiology of invasive pneumococcal disease after PCV13 vaccination in a country with intermediate vaccination coverage. *Vaccine* **36**, 7744–7752 (2018).

18. Redin, A. *et al.* Serotypes and Clonal Composition of Streptococcus pneumoniae Isolates Causing IPD in Children and Adults in Catalonia before 2013 to 2015 and after 2017 to 2019 Systematic Introduction of PCV13. *Microbiol Spectr* **9**, (2021).

19. Amin-Chowdhury, Z. *et al.* Characteristics of Invasive Pneumococcal Disease Caused by Emerging Serotypes after the Introduction of the 13-Valent Pneumococcal Conjugate Vaccine in England: A Prospective Observational Cohort Study, 2014-2018. *Clinical Infectious Diseases* **71**, E235–E243 (2020).

20. Gutiérrez Rodríguez angelesgutierrez, M. A. *et al. Surveillance and outbreak reports*. *Euro Surveill* vol. 19 www.eurosurveillance.org:pii=20922.Availableonline:http://www.eurosurveillance.org/ViewArticle.aspx?ArticleId=20922 (2008).

21. Horácio, A. N., Diamantino-Miranda, J., Aguiar, S. I., Ramirez, M. & Melo-Cristino, J. The Majority of Adult Pneumococcal Invasive Infections in Portugal Are Still Potentially Vaccine Preventable in Spite of Significant Declines of Serotypes 1 and 5. *PLoS One* **8**, (2013).

22. Horácio, A. N. *et al.* Population structure of Streptococcus pneumoniae causing invasive disease in adults in Portugal before PCV13 availability for adults: 2008-2011. *PLoS One* **11**, (2016).

23. Polkowska, A. *et al.* Pneumococcal meningitis before the introduction of 10-valent pneumococcal conjugate vaccine into the National Childhood Immunization Program in Poland. *Vaccine* **37**, 1365–1373 (2019).

24. Alari, A. *et al.* Impact of pneumococcal conjugate vaccines on pneumococcal meningitis cases in France between 2001 and 2014: A time series analysis. *BMC Med* **14**, (2016).

25. Wagenvoort, G. H. J. *et al.* Risk and outcomes of invasive pneumococcal disease in adults with underlying conditions in the post-PCV7 era, The Netherlands. *Vaccine* **34**, 334–340 (2016).

26. Ouldali, N. *et al.* Invasive pneumococcal disease prevalence in children and adults in France during the pneumococcal conjugate vaccine era: an interrupted time-series analysis of data from a 17-year national prospective surveillance study. *Lancet Infect Dis* **21**, 137–147 (2021).

27. Bahrs, C. *et al.* A longitudinal analysis of pneumococcal vaccine serotypes in pneumonia patients in Germany. *European Respiratory Journal* **59**, (2022).

28. Monali, R. *et al.* Impact of vaccination on invasive pneumococcal disease in Italy 2007-2017: Surveillance challenges and epidemiological changes. *Epidemiol Infect* (2020) doi:10.1017/S0950268820001077.

29. Lawrence, H. *et al.* Effectiveness of the 23-valent pneumococcal polysaccharide vaccine against vaccine serotype pneumococcal pneumonia in adults: A case-control test-negative design study. *PLoS Med* **17**, (2020).

30. Pick, H. *et al.* Pneumococcal serotype trends, surveillance and risk factors in UK adult pneumonia, 2013-18. *Thorax* **75**, 38–49 (2020).

31. Imöhl, M. *et al.* Pneumococcal meningitis and vaccine effects in the era of conjugate vaccination: Results of 20 years of nationwide surveillance in Germany. *BMC Infect Dis* **15**, (2015).

32. Adler, H. *et al.* Pneumococcal colonization in healthy adult research participants in the conjugate vaccine era, United Kingdom, 2010-2017. *Journal of Infectious Diseases* **219**, 1989–1993 (2019).

33. van der Linden, M., Falkenhorst, G., Perniciaro, S. & Imöhl, M. Effects of infant pneumococcal conjugate vaccination on serotype distribution in invasive pneumococcal disease among children and adults in germany. *PLoS One* **10**, (2015).

34. Skoczyńska, A. *et al.* Recent trends in epidemiology of invasive pneumococcal disease in Poland. *European Journal of Clinical Microbiology and Infectious Diseases* **34**, 779–787 (2015).

35. Valdarchi, C. *et al.* Pneumococcal carriage among adults aged 50 years and older with co-morbidities attending medical practices in Rome, Italy. *Vaccine* **37**, 5096–5103 (2019).

36. Petrović, V. *et al.* Streptococcus pneumoniae serotype distribution in Vojvodina before the introduction of pneumococcal conjugate vaccines into the National Immunization Program. *Srp Arh Celok Lek* **144**, 521–526 (2016).

37. Moore, C. E. *et al.* Reduction of invasive pneumococcal disease 3 years after the introduction of the 13-valent conjugate vaccine in the Oxfordshire region of England. *Journal of Infectious Diseases* **210**, 1001–1011 (2014).

38. Hughes, G. J., Wright, L. B., Chapman, K. E., Wilson, D. & Gorton, R. Serotype-specific differences in short- and longer-term mortality following invasive pneumococcal disease. *Epidemiol Infect* **144**, 2654–2669 (2016).

39. Waight, P. A. *et al.* Effect of the 13-valent pneumococcal conjugate vaccine on invasive pneumococcal disease in England and Wales 4 years after its introduction: An observational cohort study. *Lancet Infect Dis* **15**, 535–543 (2015).

40. Lamb, K. E. *et al.* Trends in serotypes and sequence types among cases of invasive pneumococcal disease in Scotland, 1999-2010. *Vaccine* **32**, 4356–4363 (2014).

41. Corcoran, M. *et al.* The epidemiology of invasive pneumococcal disease in older adults in the post-PCV era. Has there been a herd effect? *Epidemiol Infect* **145**, 2390–2399 (2017).

42. Bijlsma, M. W. *et al.* Community-acquired bacterial meningitis in adults in the Netherlands, 2006-14: A prospective cohort study. *Lancet Infect Dis* **16**, 339–347 (2016).

43. Vissers, M. *et al.* Increased carriage of non-vaccine serotypes with low invasive disease potential four years after switching to the 10-valent pneumococcal conjugate vaccine in The Netherlands. *PLoS One* **13**, (2018).

44. van Werkhoven, C. H. *et al.* Pneumococcal conjugate vaccine herd effects on non-invasive pneumococcal pneumonia in elderly. *Vaccine* **34**, 3275–3282 (2016).

45. Benfield, T. *et al.* Serotype Distribution in Non-Bacteremic Pneumococcal Pneumonia: Association with Disease Severity and Implications for Pneumococcal Conjugate Vaccines. *PLoS One* **8**, (2013).

46. Slotved, H. C., Dalby, T. & Hoffmann, S. The effect of pneumococcal conjugate vaccines on the prevalence of invasive pneumococcal disease caused by ten non-vaccine serotypes in Denmark. *Vaccine* **34**, 769–774 (2016).

47. Naucler, P. *et al.* Comparison of the Impact of Pneumococcal Conjugate Vaccine 10 or Pneumococcal Conjugate Vaccine 13 on Invasive Pneumococcal Disease in Equivalent Populations. *Clinical Infectious Diseases* **65**, 1780–1789 (2017).

48. Yahiaoui, R. Y. *et al.* Distribution of serotypes and patterns of antimicrobial resistance among commensal Streptococcus pneumoniae in nine European countries. *BMC Infect Dis* **18**, (2018).

49. Skoczyńska, A. *et al.* Recent trends in epidemiology of invasive pneumococcal disease in Poland. *European Journal of Clinical Microbiology and Infectious Diseases* **34**, 779–787 (2015).

50. Galanis, I. *et al.* Effects of PCV7 and PCV13 on invasive pneumococcal disease and carriage in Stockholm, Sweden. *European Respiratory Journal* **47**, 1208–1218 (2016).

51. Horácio, A. N., Lopes, J. P., Ramirez, M. & Melo-Cristino, J. Non-invasive pneumococcal pneumonia in Portugal - Serotype distribution and antimicrobial resistance. *PLoS One* **9**, (2014).

52. Aguinagalde, L. *et al.* Emergence of amoxicillin-resistant variants of Spain9V-ST156 pneumococci expressing serotype 11A correlates with their ability to evade the host immune response. *PLoS One* **10**, (2015).

53. Payeras, A. *et al.* Pneumococcal infections in elderly patients attending hospital since PCV-13 authorization in Spain. *Infect Dis* **49**, 71–80 (2017).

54. Navarro-Torné, A. *et al.* Risk factors for death from invasive pneumococcal disease, europe, 2010. *Emerg Infect Dis* **21**, 417–425 (2015).

55. Imöhl, M. *et al.* Pneumococcal meningitis and vaccine effects in the era of conjugate vaccination: Results of 20 years of nationwide surveillance in Germany. *BMC Infect Dis* **15**, (2015).

56. Richter, L. *et al.* Invasive pneumococcal diseases in children and adults before and after introduction of the 10-valent pneumococcal conjugate vaccine into the Austrian national immunization program. *PLoS One* **14**, (2019).

57. Almeida, S. T. *et al.* Low prevalence of pneumococcal carriage and high serotype and genotype diversity among adults over 60 years of age living in Portugal. *PLoS One* **9**, (2014).

58. Littorin, N., Ahl, J., Uddén, F., Resman, F. & Riesbeck, K. Reduction of Streptococcus pneumoniae in upper respiratory tract cultures and a decreased prevalence of related acute otitis media following introduction of childhood pneumococcal conjugate vaccines in a Swedish county. *BMC Infect Dis* **16**, (2016).

59. Quirk, S. J. *et al.* Vaccination of Icelandic Children with the 10-Valent Pneumococcal Vaccine Leads to a Significant Herd Effect among Adults in Iceland. *J Clin Microbiol* **57**, (2019).

60. Hyams, C. *et al.* Streptococcus Pneumoniae septic arthritis in adults in Bristol and Bath, United Kingdom, 2006–2018: a 13-year retrospective observational cohort study. *Emerg Microbes Infect* **10**, 1369–1377 (2021).

61. Verhaegen, J. *et al.* Epidemiology and outcome of invasive pneumococcal disease among adults in Belgium, 2009–2011. *Eurosurveillance* **19**, (2014).

62. van der Linden, M., Perniciaro, S. & Imöhl, M. Increase of serotypes 15A and 23B in IPD in Germany in the PCV13 vaccination era. *BMC Infect Dis* **15**, (2015).

63. Weinberger, D. M. *et al.* Differences in the impact of pneumococcal serotype replacement in individuals with and without underlying medical conditions. *Clinical Infectious Diseases* **69**, 100–106 (2019).

64. Amin-Chowdhury, Z. *et al.* Invasive pneumococcal disease due to 22F and 33F in England: A tail of two serotypes. *Vaccine* **39**, 1997–2004 (2021).

65. Siira, L. *et al.* Antimicrobial resistance in relation to sero-and genotypes among invasive streptococcus pneumoniae in Finland, 2007-2011. *Microbial Drug Resistance* **20**, 124–130 (2014).

66. Siira, L., Vestrheim, D. F., Winje, B. A., Caugant, D. A. & Steens, A. Antimicrobial susceptibility and clonality of Streptococcus pneumoniae isolates recovered from invasive disease cases during a period with changes in pneumococcal childhood vaccination, Norway, 2004–2016. *Vaccine* **38**, 5454–5463 (2020).

Supplementary table S5 - Summary of the findings from the post-PCV10/13 era (2016-2022, unless stated otherwise by the authors of each paper).

	18-64yo (IPD)	≥65yo (IPD)	18-64yo (other)	≥65yo (other)	Severity and Lethality	Antimicrobial resistance
**8**	• 15.1% prevalence in Spain [1]		• 1.6% of all-cause CAP in Germany [2]		• Associated with empyema in IPD in the Netherlands 51.1% [3]*	
	• 7% prevalence in CAP in Spain, 20.1% prevalence in invasive CAP cases [4]					
	• 30% prevalence in Spain [5]	• 19% prevalence in Spain [5]	• 7% prevalence in CAP in Spain, 7.1% prevalence in non-invasive CAP cases [4]		• Significantly associated with lower CFR (IPD, Netherlands) [3]*	
	• 18.5% prevalence in Catalonia, Spain (vs 8.1% in the PCV10/13 era) [6]*					
	• Among the most prevalent serotypes in the PCV10/13 and post-PCV10/13 era but significant increase in the latter in Catalonia, Spain [7]	• Among the most prevalent serotypes in the PCV10/13 and post-PCV10/13 era but significant increase in the latter in Catalonia, Spain [7]	• 8.1% prevalence in CAP in England - 2nd most prevalent serotype [8]		• Most frequent serotype in pneumonia cases in Spain (IPD) 16.7% [1]<br>• Significantly lower odds of meningitis (OR 0.69, IPD, England and Wales) [9]*	
		• Significant increase in prevalence vs the PCV10/13 era (Catalonia, Spain) [6]	• 18.3% prevalence in CAP in the UK [10]		• Significantly associated with increased odds of death (OR 2.9, CFR 33%) (IPD, England and Wales) [9]*	
		• 6.9% prevalence in France [12]	• Identified in 7.7% of the young adults who were carriers in the UK [11]			
	• 22.1% prevalence in Italy (2017) [13]*				• Most common in sepsis not associated with other clinical conditions (IPD, Italy) 11.6% [14]	
	• Among the most frequent serotypes in Italy 8.5% (+5.5% vs PCV7 era) [14]					
	• 21.1% of bacteremic pneumococcal CAP in Germany [2]					
	• 0.32 per 100.000 prevalence in Austria (rank 4) [15]				• Among the most prevalent serotypes (>=1% of total cases) in the presence of underlying conditions (CAP, Spain) [4]	
	• 30.3% prevalence (15-44yo), 25.7% prevalence (45-64yo) in England [16]	• 18.6% prevalence (65-79yo), 10.0% prevalence (>80yo) in England [16]		• Identified in 16.7% of the older adults who were carriers in Italy [17]		
	• 27.2% prevalence in England and Wales, increased vs PCV10/13 [18][$]	• 15.7% prevalence in England and Wales, increased vs PCV10/13 [18]			• Most prevalent serotype in patients with chronic renal failure, patients with asthma, tobacco cigarette smokers, and patients with diabetes mellitus (33.3%, 14.6%, 14.2%, 8.7%) (CAP, Spain) [4]	
	• Predominant cause of meningitis in England and Wales (2015-16) [9][$]	• 25% of the cases of meningitis in England and Wales (2015-16) [9]				
	• Significantly increased vs PCV10/13 era IRR 1.18 (North East England) [19]*				• Among the serotypes most associated with risk of sepsis/septic shock on admission or during hospitalization OR 4.9 (CAP, Spain) [4]	
		• 2.31 per 100.000 prevalence in Ireland (2015-2016), significant vs PCV7 IRR 3.63 [20]				
	• Prevalence in the Netherlands 3.7 per 100.000 (significant increase from 2010) [3]*					

	18-64yo (IPD)	≥65yo (IPD)	18-64yo (other)	≥65yo (other)	Severity and Lethality	Antimicrobial resistance
		• 11.3 per 100.000 prevalence in the Netherlands, steep increase vs PCV10/13 era [21]			• Higher prevalence in younger adults than older (15-64 vs ≥65yo, IPD, England) [16]	
	• Significantly increasing vs PCV10/13 era in IPD in Denmark (IRR ~2) [22]				• More present in individuals with no comorbidities comparing to PCV13 serotypes (IPD, England) [16]	
	• 0.75 per 100.000 prevalence in Sweden (2016), 5th most prevalent [23]*					
		• 17% prevalence in 2018 across several countries in Europe vs 8% in 2012 (Czech Republic, Denmark, England, Finland, France, Ireland, the Netherlands, Norway, Scotland, Sweden, Spain) [24]			• Significantly lower CFR comparing to PCV13 serotypes OR 0.44 (IPD, England) [16]	
**10A**	• 2.3% prevalence in Spain [5]	• 1.8% prevalence in Spain [5]			• Significantly higher odds of meningitis (OR 2.06, IPD, England and Wales) [9]*	
	• 2.3% prevalence in Catalonia, Spain [6]*					
		• 3.0% prevalence in France [12]				
	• 3.6% prevalence in Italy (2017) [13]*					
		• 5.4% prevalence (>50yo) in Hungary [25]				
	• 2.5% prevalence in England and Wales [18]$	• 3.1% prevalence in England and Wales [18]				
	• 0.46 per 100.000 prevalence in Sweden (2016) [23]*					
**11A**	• 2.9% prevalence in Spain [5]	• 3.3% prevalence in Spain [5]	• 0.8% of all-cause CAP in Germany [2]		• Significantly associated with higher CFR (IPD, Netherlands) [3]*	• Clonal replacement linked to an increase in PEN+AMOX-resistant isolates in IPD in Spain, France, Portugal, and Italy – 0 resistant isolates in 2008 vs 11 out of 13 in 2016 [26]
	• 0.36 per 100.000 prevalence in Spain (non-significant increase vs PCV7 era) [26]		• 3.5% prevalence in CAP in the UK [10]		• Most frequent serotype in peritonitis cases in Spain (IPD) 19% [1]	
	•					
	• 2.6% prevalence in Catalonia, Spain [6]*					
		• Significant increase in prevalence vs the PCV10/13 era (Catalonia, Spain) [6]	• Identified in 7.7% of the young adults who were carriers in the UK [11]		•	
	• 1.8% prevalence in Italy (2017) [13]*					
	• 0.18 per 100.000 prevalence in Austria (rank 10) [15]					• β-lactam-resistance associated with serotype 11A (IPD, Spain) [1]
	• 7.4% prevalence (7-49yo), 14.3% prevalence (>50yo) in Hungary [25]					
	• 1.6% prevalence in England and Wales [18]$	• 2.4% prevalence in England and Wales [18]		• Identified in 8.3% of the older adults who were		• Clone Spain9V-ST156, mainly expressing
		• 0.66 per 100.000 prevalence in Ireland (2015-2016) [20]				

	18-64yo (IPD)	≥65yo (IPD)	18-64yo (other)	≥65yo (other)	Severity and Lethality	Antimicrobial resistance
	• 0.46 per 100.000 prevalence in Sweden (2016) [23]*			carriers in Italy [17]		serotype 11A, accounted for more than 50% of IPD episodes caused by penicillin-non-susceptible pneumococci after PCV13 [27]
	• 7.1 per 100.000 prevalence in Iceland [28]	• 27.8 per 100.000 prevalence in Iceland [28]				
**12F**	• 7.9% prevalence in Spain [1]		• 2.5% prevalence in CAP in England – 3rd most prevalent serotype [8]		• Among the most frequent serotypes in septic arthritis (UK) (6 out of 21 cases) [29]<br>• Significantly associated with lower CFR (IPD, Netherlands) [3]*	
	• 6.3% prevalence in Spain [5]	4.6% prevalence in Spain [5]				
	• 8.0% prevalence in Catalonia, Spain (vs 8.1% in the PCV10/13 era) [6]*		• 5.6% prevalence in CAP in the UK [10]			
		• Significant increase in prevalence vs the PCV10/13 era (Catalonia, Spain) [6]			• Among the most prevalent serotypes (>=1% of total cases) in the presence of underlying conditions (CAP, Spain) [4]	
	• Among the most prevalent serotypes in the PCV10/13 and post-PCV10/13 era but significant increase in the latter in Catalonia, Spain [7]				• Among the serotypes most associated with risk of ICU admission on admission or during hospitalization OR 3.5 (CAP, Spain) [4]	
		• 4.8% prevalence in France [12]				
	• 10.1% prevalence in Italy (2016) [13]*				• Higher prevalence in younger adults than older (15-64 vs ≥65yo, IPD, England) [16]	
	• 5.3% of bacteremic pneumococcal CAP in Germany [2]				• More present in individuals with no comorbidities comparing to PCV13 serotypes (IPD, England) [16]	
	• 19.4% prevalence (15-44yo), 15.4% prevalence (45-64yo) in England [16]	• 9.4% prevalence (65-79yo), 6.4% prevalence (>80yo) in England [16]				
	• Significantly increased vs PCV10/13 era IRR 1.28 (North East England) [19]*				• Significantly lower CFR comparing to PCV13 serotypes OR 0.54 (IPD, England) [16]	
	• 17.9% prevalence in England and Wales, increased vs PCV10/13 [18]$	• 9.3% prevalence in England and Wales, increased vs PCV10/13 [18]				
	• Predominant cause of meningitis in England and Wales (2015-16) [9]$					
		• 2.48 per 100.000 prevalence in Ireland (2015-2016) [20]				
	• 0.66 per 100.000 prevalence in Sweden (2016), 6th most prevalent [23]*					
**15B**	• 1.2% prevalence in Spain [5]	• 2.4% prevalence in Spain [5]		• Identified in 8.3% of the older adults	• Significantly associated with higher CFR (IPD, Netherlands) [3]*	
		• 2.4% prevalence in France [12]&				
	• 2.0% prevalence in Italy (2017) [13]*					

	18-64yo (IPD)	≥65yo (IPD)	18-64yo (other)	≥65yo (other)	Severity and Lethality	Antimicrobial resistance
	• 1.2% prevalence in England and Wales [18]$&	• 1.5% prevalence in England and Wales [18]&		who were carriers in Italy [17]		
	• 5.8 per 100.000 prevalence in Iceland [28]&	• 6.2 per 100.000 prevalence in Iceland [28]&				
	• 0.20 per 100.000 prevalence in Sweden (2016) [23]*					
**22F**	• 4.1% prevalence in Spain [1]		• 1.0% of all-cause CAP in Germany [2]		• Among the most frequent serotypes in septic arthritis (UK) [29]	
	• 4.6% prevalence in Spain [5]	5.3% prevalence in Spain [5]				
	• 4.5% prevalence in Catalonia, Spain (vs 3.6% in the PCV10/13 era) [6]*		• 3.3% prevalence in CAP in the UK [10]		• Associated with empyema in IPD in the Netherlands 13.3% [3]*	
		• 8.1% prevalence in France [12]				
	• 5.9% prevalence in Italy (2017) [13]*				• Significantly higher odds of meningitis (OR 1.39, IPD, England and Wales) [9]*	
	• 5.1% prevalence in Italy (decreasing over time and not among the most common) [14]					
	• 0.45 per 100.000 prevalence in Austria (rank 3) [15]				• Higher prevalence in older adults than younger age groups, particularly ≥80 yo (IPD, England) [30]	
	• 7.0% prevalence in England [30]					
	• Increased in prevalence after PCV7 era, plateaued in PCV10/13 era (England) [30]					
	• 16.5% prevalence in England [16]*				• Comorbidity prevalence of 68.7% - significantly higher than other serotypes altogether (IPD, England) [30]	
	• 6.2% prevalence in England and Wales, plateaued vs PCV10/13 era [18]$	• 7.3% prevalence in England and Wales, plateaued vs PCV10/13 era [18]				
		• 2.15 per 100.000 prevalence in Ireland (2015-2016) [20]			• CFR 15.4% - significantly lower than other serotypes altogether (IPD, England) [30]	
		• 2.9 per 100.000 prevalence in the Netherlands, relatively stable vs PCV10/13 era [21]			• Death in 30d in patients with the 22F serotype associated with increasing age and clinical presentation with meningitis or septicaemia vs invasive pneumonia [30]	
	• 1.44 per 100.000 prevalence in Sweden (2016), 2nd most prevalent [23]*					
	• Caused most of the cases of pneumococcal meningitis in Finland 25% [31]*					
	• 3.2 per 100.000 prevalence in Iceland [28]&	• 15.4 per 100.000 prevalence in Iceland [28]&			• Among the serotypes most associated with risk of sepsis/septic shock on admission or during hospitalization OR 4.5 (CAP, Spain) [4]	
		• 7% prevalence in 2018 across several countries in Europe (Czech Republic, Denmark, England, Finland, France, Ireland, the Netherlands, Norway, Scotland, Sweden, Spain) [24]				
**33F**	• 2.2% prevalence in Spain [5]	• 3.2% prevalence in Spain [5]			• Highest prevalence in adults aged 65-79 (IPD, England) [30]	
	• 2.5% prevalence in Catalonia, Spain (vs 1.6% in the PCV10/13 era) [6]*				• Comorbidity prevalence of 67.2% - significantly higher than other serotypes altogether (IPD, England) [30]	
		• Significant increase vs PCV10/13 in Catalonia, Spain [7]				
	• 1.5% prevalence in Italy (2017) [13]*					

	18-64yo (IPD)	≥65yo (IPD)	18-64yo (other)	≥65yo (other)	Severity and Lethality	Antimicrobial resistance
	• 5.3% of bacteremic pneumococcal CAP in Germany [2]				• CFR 16.5% - significantly lower than other serotypes altogether (IPD, England) [30]	
	• 3.5% prevalence in England [30]				• Death in 30d in patients with the 33F serotype associated with increasing age, comorbidity status and clinical presentation with septicaemia vs invasive pneumonia [30]	
	• Increased in prevalence after PCV7 era, plateaued in PCV10/13 era (England) [30]					
	• 8.6% prevalence in England [16]*					
		• 2.31 per 100.000 prevalence in Ireland (2015-2016) [20]				
	• 2.5% prevalence in England and Wales [18]$	• 3.6% prevalence in England and Wales [18]				
	• 0.38 per 100.000 prevalence in Sweden (2016) [23]*					
	• 1.3 per 100.000 prevalence in Iceland [28]					

* total population, $ 5-64 years, & 15B/C

AMOX amoxicillin, CFR case fatality ratio, IRR prevalence rate ratio, MIC minimal inhibitory concentration, OR odds ratio, PEN penicillin

References

1. González-Díaz, A. *et al.* Emerging non-13-valent pneumococcal conjugate vaccine (PCV13) serotypes causing adult invasive pneumococcal disease in the late-PCV13 period in Spain. *Clinical Microbiology and Infection* **26**, 753–759 (2020).
2. Bahrs, C. *et al.* A longitudinal analysis of pneumococcal vaccine serotypes in pneumonia patients in Germany. *European Respiratory Journal* **59**, (2022).
3. Vestjens, S. M. T. *et al.* Twelve years of pneumococcal conjugate vaccination in the Netherlands: Impact on prevalence and clinical outcomes of invasive pneumococcal disease. *Vaccine* **37**, 6558–6565 (2019).
4. Torres, A. *et al.* The Evolution and Distribution of Pneumococcal Serotypes in Adults Hospitalized With Community-Acquired Pneumonia in Spain Using a Serotype-Specific Urinary Antigen Detection Test: The CAPA Study, 2011-2018. *Clin Infect Dis* **73**, 1075–1085 (2021).
5. de Miguel, S. *et al.* Nationwide Trends of Invasive Pneumococcal Disease in Spain From 2009 Through 2019 in Children and Adults During the Pneumococcal Conjugate Vaccine Era. *Clinical Infectious Diseases* **73**, e3778–e3787 (2021).
6. Redin, A. *et al.* Serotypes and Clonal Composition of Streptococcus pneumoniae Isolates Causing IPD in Children and Adults in Catalonia before 2013 to 2015 and after 2017 to 2019 Systematic Introduction of PCV13. *Microbiol Spectr* **9**, (2021).
7. Ludwig, G. *et al.* Serotype and clonal distribution dynamics of invasive pneumococcal strains after PCV13 introduction (2011-2016): Surveillance data from 23 sites in Catalonia, Spain. *PLoS One* **15**, (2020).
8. Lawrence, H. *et al.* Effectiveness of the 23-valent pneumococcal polysaccharide vaccine against vaccine serotype pneumococcal pneumonia in adults: A case-control test-negative design study. *PLoS Med* **17**, (2020).
9. Oligbu, G. *et al.* Effect of pneumococcal conjugate vaccines on pneumococcal meningitis, England and Wales, July 1, 2000-June 30, 2016. *Emerg Infect Dis* **25**, 1708–1718 (2019).
10. Pick, H. *et al.* Pneumococcal serotype trends, surveillance and risk factors in UK adult pneumonia, 2013-18. *Thorax* **75**, 38–49 (2020).
11. Adler, H. *et al.* Pneumococcal colonization in healthy adult research participants in the conjugate vaccine era, United Kingdom, 2010-2017. *Journal of Infectious Diseases* **219**, 1989–1993 (2019).

12. Ouldali, N. *et al.* Invasive pneumococcal disease prevalence in children and adults in France during the pneumococcal conjugate vaccine era: an interrupted time-series analysis of data from a 17-year national prospective surveillance study. *Lancet Infect Dis* **21**, 137–147 (2021).

13. Monali, R. *et al.* Impact of vaccination on invasive pneumococcal disease in Italy 2007-2017: Surveillance challenges and epidemiological changes. *Epidemiol Infect* (2020) doi:10.1017/S0950268820001077.

14. Nieddu, F. *et al.* PCV13 serotype decrease in Italian adolescents and adults in the post-PCV13 era: Herd protection from children or secular trend? *Vaccine* **35**, 1544–1550 (2017).

15. Richter, L. *et al.* Invasive pneumococcal diseases in children and adults before and after introduction of the 10-valent pneumococcal conjugate vaccine into the Austrian national immunization program. *PLoS One* **14**, (2019).

16. Amin-Chowdhury, Z. *et al.* Characteristics of Invasive Pneumococcal Disease Caused by Emerging Serotypes after the Introduction of the 13-Valent Pneumococcal Conjugate Vaccine in England: A Prospective Observational Cohort Study, 2014-2018. *Clinical Infectious Diseases* **71**, E235–E243 (2020).

17. Valdarchi, C. *et al.* Pneumococcal carriage among adults aged 50 years and older with co-morbidities attending medical practices in Rome, Italy. *Vaccine* **37**, 5096–5103 (2019).

18. Ladhani, S. N. *et al.* Rapid increase in non-vaccine serotypes causing invasive pneumococcal disease in England and Wales, 2000–17: a prospective national observational cohort study. *Lancet Infect Dis* **18**, 441–451 (2018).

19. Houseman, C., Hughes, G. J., Chapman, K. E., Wilson, D. & Gorton, R. Increased invasive pneumococcal disease, North East England, UK. *Emerg Infect Dis* **23**, 122–126 (2017).

20. Corcoran, M. *et al.* The epidemiology of invasive pneumococcal disease in older adults in the post-PCV era. Has there been a herd effect? *Epidemiol Infect* **145**, 2390–2399 (2017).

21. Vissers, M. *et al.* Increased carriage of non-vaccine serotypes with low invasive disease potential four years after switching to the 10-valent pneumococcal conjugate vaccine in The Netherlands. *PLoS One* **13**, (2018).

22. Hansen, C. B. *et al.* Molecular characterization and epidemiology of Streptococcus pneumoniae serotype 8 in Denmark. *BMC Infect Dis* **21**, (2021).

23. Naucler, P. *et al.* Comparison of the Impact of Pneumococcal Conjugate Vaccine 10 or Pneumococcal Conjugate Vaccine 13 on Invasive Pneumococcal Disease in Equivalent Populations. *Clinical Infectious Diseases* **65**, 1780–1789 (2017).

24. Hanquet, G. *et al.* Serotype Replacement after Introduction of 10-Valent and 13-Valent Pneumococcal Conjugate Vaccines in 10 Countries, Europe. *Emerg Infect Dis* **28**, 127–138 (2022).

25. Kovács, E. *et al.* Vaccine-driven serotype-rearrangement is seen with latency in clinical isolates: Comparison of carried and clinical pneumococcal isolates from the same time period in Hungary. *Vaccine* **37**, 99–108 (2019).

26. González-Díaz, A. *et al.* Two multi-fragment recombination events resulted in the β-lactam-resistant serotype 11A-ST6521 related to Spain9V-ST156 pneumococcal clone spreading in south-western Europe, 2008 to 2016. *Eurosurveillance* **25**, (2020).

27. Càmara, J. *et al.* A historical perspective of MDR invasive pneumococcal disease in Spanish adults. *Journal of Antimicrobial Chemotherapy* **76**, 507–515 (2021).

28. Quirk, S. J. *et al.* Vaccination of Icelandic Children with the 10-Valent Pneumococcal Vaccine Leads to a Significant Herd Effect among Adults in Iceland. *J Clin Microbiol* **57**, (2019).

29. Hyams, C. *et al.* Streptococcus Pneumoniae septic arthritis in adults in Bristol and Bath, United Kingdom, 2006–2018: a 13-year retrospective observational cohort study. *Emerg Microbes Infect* **10**, 1369–1377 (2021).

30. Amin-Chowdhury, Z. *et al.* Invasive pneumococcal disease due to 22F and 33F in England: A tail of two serotypes. *Vaccine* **39**, 1997–2004 (2021).

31. Polkowska, A., Rinta-Kokko, H., Toropainen, M., Palmu, A. A. & Nuorti, J. P. Long-term population effects of infant 10-valent pneumococcal conjugate vaccination on pneumococcal meningitis in Finland. *Vaccine* **39**, 3216–3224 (2021).

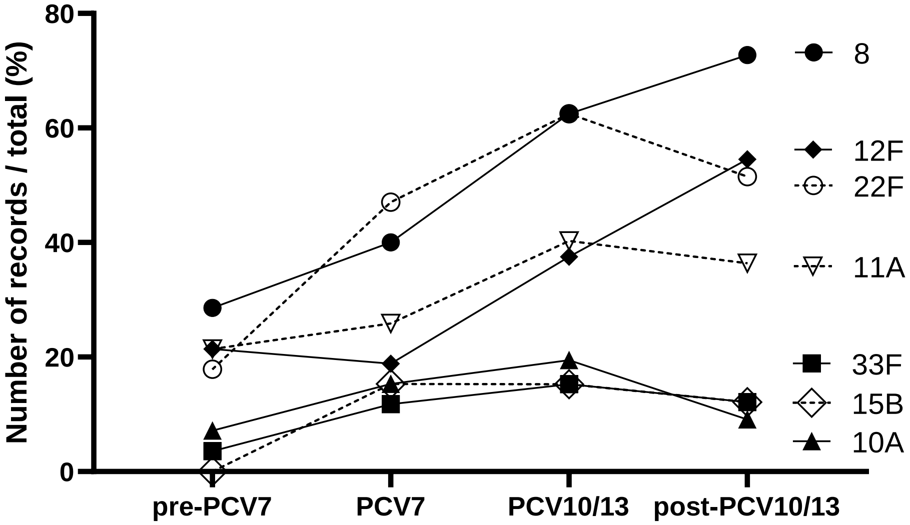

Figure S1 – Number of records included in the study for each serotype for each PCV period divided by the total number of records considered for each PCV era (%). Serotypes were included if they were central in the study, i.e., amongst the most prevalent, representing more serious pneumococcal disease or showing antimicrobial resistance. Articles spanning several PCV eras (e.g., PCV7 and PCV10/13) were counted for the total of records in all time periods that applied.
